# Supplementary material for: Differential DNA methylation of vocal and facial anatomy genes in modern humans
Source: Nat Commun. 2020 Mar 4;11:1189. doi: 10.1038/s41467-020-15020-6 (PMC7055320; doi:10.1038/s41467-020-15020-6)
Supplement: Supplementary file 1 — Supplementary Information [file 41467_2020_15020_MOESM1_ESM.pdf]

Supplementary Information for the paper:

Genes Affecting Vocal and Facial Anatomy Went Through Extensive Methylation Changes in Modern Humans

Gokhman et al.

Corresponding authors: David Gokhman (david.gokhman@mail.huji.ac.il), Eran Meshorer (eran.meshorer@mail.huji.ac.il), and Liran Carmel (liran.carmel@huji.ac.il)

Supplementary Methods

Skeletal Methylation Maps

Previously, our ability to identify differentially methylated regions (DMRs) that discriminate between human groups was confined by three main factors: (i) We had a single DNA methylation map from a present-day human bone, which was produced using a reduced representation bisulfite sequencing (RRBS) protocol, which provides information for only ~10% of CpG positions in the genome. Moreover, the fact that the archaic and present-day methylomes were produced using different technologies – computational reconstruction versus RRBS – potentially introduces a bias. (ii) The analyses included only one bone methylation map from each of the human groups, which limited our ability to identify fixed differences between the groups. Although dozens of maps from additional tissues in present-day humans were included in the analyses, this narrowed the DMRs to represent only human-specific changes that are invariable between tissues. (iii) The work did not include a great ape outgroup. Thus, when a AMH-specific change was identified, it was impossible to determine whether it happened on the AMH lineage, or in the ancestor of Neanderthals and Denisovans[1].

To overcome these obstacles, a major goal of the current study was to significantly extend the span of our skeletal methylome collection, covering as many individuals, sexes, and bone types as we could. This included the generation of many samples, including the high-coverage sequencing of additional ancient genomes, as listed below.

Present-day human bone DNA methylation maps

We generated full DNA methylation maps from two femur head bones from present-day humans using whole-genome bisulfite sequencing (WGBS). Femora were chosen because of their

abundance in present-day human samples, as well as in ancient DNA samples[2–4]. In addition, we collected 53 publicly available partial skeletal methylation maps.

Trabecular bone tissue from femur heads were taken from two patients with osteoarthritis during a total hip replacement surgery, and after filling in a consent form as per Helsinki approval #0178-13-HMO. Importantly, the effects of osteoarthritis processes on trabecular bone are much less substantial than those on the synovium, cartilage, and subchondral bone. Bone1 was a left head of femur taken on August 11, 2014 from a 66 years old female and Bone 2 was a right head of femur taken on September 2, 2014 from a 63 years old female.

DNA was extracted from bones using QIAamp® DNA Investigator kit (56504, Qiagen). Bones were cut to thin slices (0.2-0.5 mm) and then thoroughly washed (X5) with PBS, to clean samples from blood. Bones were crushed with mortar and pestle in liquid nitrogen, and 100 mg bone powder was taken to extract DNA according to the protocol Isolation of Total DNA from Bones and Teeth of the DNA Investigator kit.

Whole-genome bisulfite sequencing was conducted at the Centre Nacional d'analysis Genomico (CNAG) as described in[5]. After cell sorting, genomic DNA libraries were constructed using the Illumina TruSeq Sample Preparation kit (Illumina) following the manufacturer's standard protocol. DNA was then exposed to two rounds of sodium bisulfite treatment using the EpiTect Bisulfite kit (QIAGEN), and paired-end DNA sequencing was performed using the Illumina Hi-Seq 2000. We used the GEM mapper[6] with two modified versions each of the human (GRCh37) and viral reference genomes: one with all C's changed to T's and another with all G's changed to A's. Reads were fully converted in silico prior to mapping to the modified reference genomes, and the original reads were restored after mapping. Although methylation state should not depend on read position, positional biases have been previously reported[7]. We observed that the

first few bases from each read showed a slightly higher probability of being called as methylated, and we thus trimmed the first ten bases from each read (M-bias filtering).. Heterozygous positions, positions with a genotype error probability greater than 0.01, and positions with a read depth greater than 250 were filtered out. Only cytosines with six or more reads informative for methylation status were considered. On average, half of the reads from either strand will be informative for methylation status at a given position, so minimum coverage is typically greater than 12. Methylated and unmethylated cytosine conversion rates were determined from spiked-in bacteriophage DNA (fully methylated phage T7 and unmethylated phage lambda). Five samples were excluded based on conversion rates <0.997, supported by visual inspection of CG and non-CG methylation plots. The over-conversion rates for all samples based on methylated phage T7 DNA were ~5%.

Sequence quality was evaluated using FastQC software v0.11.2. TRIMMOMATIC v.0-32 was used to filter low quality bases with the following parameters: -phred33 LEADING:30 TRAILING:30 MAXINFO:70:0.9 MINLEN:70. Paired-end sequencing reads were mapped to bisulfite converted human (hg19) reference genome using Bismark v0.14.3 and bowtie2 v2.2.4 not allowing multiple alignments and using the following parameters: --bowtie2 --non_bs_mm --old_flag -p 4. Potential PCR duplicates were removed using Bismark's deduplicate_bismark_alignment_output.pl Perl program. Bismark's bismark_methylation_extractor script was used to produce methylation calls with the following parameters: -p --no_overlap --comprehensive --merge_non_CpG --no_header --bedGraph --multicore 2 --cytosine_report. Examination of the M-bias plots led us to ignore the first 5 bp of both reads in human samples (Supplementary Figure 1). Custom scripts were used to summarize methylation levels at CpG sites based on the frequencies of methylated and unmethylated

mapped reads on both strands. Methylation data were deposited in NCBI's Gene Expression Omnibus and are accessible through GEO accession number GSE96833.

Partial skeletal and full non-skeletal DNA Methylation maps

Osteoblast RRBS map, extracted from the femur, tibia, and rib bones of a 6-year-old female (NHOst-Osteoblasts by Lonza Pharma, product code: CC-2538, lot number: 6F4124), was downloaded from GEO accession number GSE27584. 48 450K methylation array maps, extracted from the femora of adult males and females with osteoarthritis or osteoporosis, were downloaded from GEO accession number GSE64490. Four 450K methylation array maps, extracted from unspecified bones of adult males and females were downloaded from GEO accession number GSE50192. Chimpanzee and human WGBS blood methylation maps were downloaded from NCBI SRA accession number SRP059313. Chimpanzee and human WGBS brain maps were downloaded from GEO accession number GSE37202.

Bisulfite-PCR of human bone

A skull of an adult male from India was obtained from the teaching anatomy collection of the Department of Anatomy and Anthropology at the Sackler Faculty of Medicine, Tel Aviv University, Israel (Human 1). Additional two skull specimens (Human 2 and 3) were obtained directly from the operating room of the Department of Neurosurgery, Shaare Zedek Medical Center, Jerusalem, Israel and transferred on dry ice for further analysis. All study participants provided informed consent according to an institutional review board – approved protocol (SZMC 0048-18).

Human 1: Standard precautions to avoid contamination were taken, including wearing disposable coats, masks, hair covers and double gloves. All following steps were performed in a UV cabinet dedicated for the preparation of ancient bone samples and located in a physically separated

ancient DNA laboratory at the Faculty of Dental Medicine. The skull was cleaned with an excess of 10% bleach (equal to 0.6% Sodium hypochlorite) and then subjected to UV radiation for 30 minutes. The cortical layer on the temporal surface (*facies temporalis*) of the zygomatic bone (ZB) was removed by low-speed drilling using a Wolf Multitool Combitool Rotary Multi Purpose Tool equipped with a sterile dental burr. Another sterile burr was used to obtain powder of the subcortical trabecular bone within the body of the zygoma. The powder was collected onto a 10 x 10 cm aluminum foil sheet pretreated with a 10% bleach solution and then transferred into a sterile 1.5 ml Eppendorf tube for subsequent DNA extraction. Altogether, three samples were obtained: ZB-3 from the right zygoma weighing 20.3 mg, and ZB-3/1 and ZB-3/2 from the left zygoma weighing 29.5 mg and 30.3 mg, respectively. Bone DNA was purified from the three bone powder samples using QIAamp DNA Investigator Kit (QIAgen, 56504) according to manufacturer's instructions.

Human 2 and 3: DNA was extracted from bones using QIAamp® DNA Investigator kit (56504, Qiagen). Bones were thoroughly washed (X5) with PBS, to clean samples from blood. Bones were crushed with mortar and pestle in liquid nitrogen, and 100 mg bone powder was taken to extract DNA according to the protocol Isolation of Total DNA from Bones and Teeth of the DNA Investigator kit.

Genomic DNA was bisulfite converted with the EZ DNA Methylation – Lightning Kit (Zymo Research, D5030) according to the manufacturer's instructions. Specifically, each bone sample was bisulfite converted using 500ng as genomic DNA input for the conversion.

Bisulfite treated DNA were amplified with the FastStart High Fidelity PCR System (Sigma, 03553400001) using the primers listed in Supplementary Table 1. PCR conditions were performed according to manufacturer's instructions and PCR products were visualized on a 1.5

% agarose gel. Prior to cloning, PCR products were purified with Gel/PCR DNA Mini Kit (RBC, YDF100) and quantified with a NanoDrop 2000 spectrophotometer.

CloneJET PCR Cloning Kit (Thermo Scientific, K1231) was used to clone the purified PCR products into a pJET1.2/blunt Cloning Vector following the Blunt-End Cloning Protocol described in the manufacturer's instructions. 5µl of each cloning reaction product were used for transformation of DH5α Competent Cells (Invitrogen, 18265017). Colonies were grown overnight on LB plates containing 100µg/ml ampicillin. Positive transformants were picked and grown overnight in liquid LB medium containing 100 µg/ml ampicillin. Subsequently, plasmid minipreps were purified with a RBC Miniprep Kit (YPD100) according to manufacturer's instructions. Purified plasmids were quantified with a NanoDrop 2000 spectrophotometer and sequenced on an Applied Biosystems 3730xl Genetic Analyzer (Supplementary Figure 5a,b).

Human primary chondrocyte validation

Primary chondrocyte cultures were obtained from osteoarthritis (OA) donors in accordance with Hadassah Medical Center Institutional Review Board approval and in accordance with the Helsinki Declaration of ethical principles for medical research involving human subjects. End-stage OA patients, with a Kellgren and Lawrence OA severity score of 3-4 were recruited following receipt of a formal written informed consent (n=8; 75% female, mean age 73±7.2 years; mean body mass index 30.1 ±5.4 kg/m^2). Hyaline articular cartilage was dissected and human chondrocytes isolated using 3 mg/mL Collagenase Type II (Worthington Cat # LS004177) in DMEM medium (Sigma-Aldrich, St Louis, MI) containing 10% FCS and 1% penicillin-streptomycin (Beit-Haemek Kibutz, Israel), 37°C, 24h incubation. Isolated cells were filtered through a nylon cell strainer (40mm diameter), washed three times with PBS and plated at 1.5 million cells per 14 cm^2 tissue culture dish (passage 0, passage 2). Cells were cultured in

standard incubation conditions (37°C, 5% CO_2) until confluence. Chondrocyte DNA purification was performed using GenElute™ Mammalian Genomic DNA Miniprep Kit (Sigma, G1N350).

Chimpanzee bone DNA methylation maps

Overall, we produced six methylation maps from bones of six common chimpanzee (*Pan troglodytes*) individuals. They include one WGBS of a wild chimpanzee, one RRBS of an infant chimpanzee, and four 850K methylation arrays of captive chimpanzees.

Chimpanzee tissue samples included in this study were opportunistically collected at routine necropsy of these animals. No animals were sacrificed for this study, and no living animals were used in this study.

WGBS of a chimpanzee bone

We used a rib bone of a 47-year-old female Chimpanzee provided from the Biobank of the Biomedical Primate Research Centre (BPRC), The Netherlands. The postmortem interval was approximately 10-12 hours. The bone was collected during the necropsy procedure and immediately frozen and stored at –80 ºC.

DNA was extracted in a dedicated ancient DNA laboratory at the Institute of Evolutionary Biology in Barcelona, where no previous work on great apes has ever been conducted. Standard precautions to avoid and monitor exogenous contamination such as frequent cleaning of bench surfaces with bleach, use of sterile coveralls, UV irradiation and blank controls were taken during the process. 200 mg of bone powder were obtained by drilling and the sample was extracted following the Dabney et al. (2013) method[8]. A final 25 µL of extract volume was used for genome sequencing.

Analysis was performed similarly to Bone1 and Bone2, with the exception that the BSreads were mapped to bisulfite converted chimpanzee (panTro4) reference genome, and we ignored the first

5bp of read1 and the first 44 bp of read2 in the chimpanzee sample (Supplementary Figure 1). Methylation data were deposited in NCBI's Gene Expression Omnibus and are accessible through GEO accession number GSE96833.

RRBS of a chimpanzee bone

We used two unidentified long bone fragments that belonged to a newborn wild chimpanzee infant who died during a documented infanticide event at Gombe National Park on 9 March 2012. The infant was known to be the offspring of a chimpanzee called Eliza and was partially eaten by an adult female and her family. The sample was collected from the ground about 48 hours after the infant's death and stored in RNAlater solution until arrival at Arizona State University (ASU). At ASU the sample was stored at 4°C until extraction.

Sampling and DNA extractions were conducted at the ASU Ancient DNA Laboratory, a Class 10,000 clean-room facility in a separate building from the Molecular Anthropology Laboratory. Precautions taken to avoid contamination included bleach decontamination and UV irradiation of tools and work area before and between uses, and use of full body coverings for all researchers. The bone samples were pulverized together in December 2012 using a SPEX CertiPrep Freezer Mill. Three DNA extractions were conducted using 50-100 mg of bone powder (Supplementary Table 2) and following the extraction protocol by Rohland and Hofreiter[9]. Two extraction blank controls were included to monitor contamination of the extraction process. One µL each of the sample extract and the blank control were used for fluorometric quantification with the Qubit 2.0 Broad Range assay. All extracts were combined for a total volume of 345 µL and approximately 0.652 µg of total DNA.

RRBS libraries were generated according to Boyle *et al.*[10]. 100-200 ng genomic DNA was digested with MspI. Subsequently, the digested DNA fragments were end-repaired and

adenylated in the same reaction. After ligation with methylated adapters, samples with different adapters were pooled together and were subjected to bisulfite conversion using the EpiTect Bisulfite kit (QIAGen) per the manufacturer's recommendations with the following modification: after first bisulfite conversion, the converted DNA was treated with sodium bisulfite again to guarantee that conversion rates were no less than 99%. Two third of bisulfite converted DNA was PCR amplified and final RRBS libraries were sequenced in an Illumina HiSeq 2000 sequencer (Supplementary Data 1). Methylation data were deposited in NCBI's Gene Expression Omnibus and are accessible through GEO accession number GSE96833.

850K DNA methylation arrays

Four chimpanzee cadavers from captive colonies at the Southwest National Primate Research Center in Texas were used. Femora were opportunistically collected at routine necropsy of these animals and stored in -20°C freezers at the Texas Biomedical Research Institute after dissection. These preparation and storage conditions ensured the preservation of skeletal DNA methylation patterns.

Samples were then transported to ASU and DNA was extracted from the femoral trabecular bone using a phenol-chloroform protocol optimized for skeletal tissues [11]. From the distal femoral condyles, trabecular bone was collected using coring devices and pulverized into bone dust using a SPEX SamplePrep Freezer/Mill. Specifically, bone cores were obtained from a transverse plane through the center of the medial condyle on the right distal femur, such that the articular surface remained preserved. Cortical bone was removed from these cores using a Dremel (Supplementary Table 3). Tissue collections were performed at the Texas Biomedical Research Institute, and DNA extractions were conducted at the ASU Molecular Anthropology Laboratory.

Genome-wide DNA methylation was assessed using Illumina Infinium MethylationEPIC microarrays. These arrays analyze the methylation status of over 850,000 sites throughout the genome, covering over 90% of the sites on the Infinium HumanMethylation450 BeadChip as well as an additional 350,000 sites within enhancer regions. For each sample, 400 ng of genomic DNA was bisulfite converted using the EZ DNA Methylation™ Gold Kit according to the manufacturer's instructions (Zymo Research), with modifications described in the Infinium Methylation Assay Protocol. These protocols were conducted at the ASU Molecular Anthropology Laboratory. Following manufacturer guidelines (Illumina), this processed DNA was then whole-genome amplified, enzymatically fragmented, hybridized to the arrays, and imaged using the Illumina iScan system. These protocols were conducted at the Texas Biomedical Research Institute. These array data have been deposited in NCBI's Gene Expression Omnibus and are accessible through GEO Series accession number GSE94677.

Raw fluorescent data were normalized to account for the noise inherent within and between the arrays themselves. Specifically, we performed a normal-exponential out-of-band (Noob) background correction method with dye-bias normalization to adjust for background fluorescence and dye-based biases and followed this with a between-array normalization method (functional normalization) which removes unwanted variation by regressing out variability explained by the control probes present on the array as implemented in the minfi package in R which is part of the Bioconductor project. This method has been found to outperform other existing approaches for studies that compare conditions with known large-scale differences[12], such as those assessed in this study.

After normalization, methylation values (β values) for each site were calculated as the ratio of methylated probe signal intensity to the sum of both methylated and unmethylated probe signal

intensities. These β values range from 0 to 1 and represent the average methylation levels at each site across the entire population of cells from which DNA was extracted (0 = completely unmethylated sites, 1 = fully methylated sites).

$$\beta = \frac{\text{Methylated Signal}}{(\text{Methylated Signal} + \text{Unmethylated Signal})}$$

Every β value in the Infinium platform is accompanied by a detection *P*-value, and those with failed detection levels (*P*-value > 0.05) in greater than 10% of samples were removed from downstream analyses.

The probes on the arrays were designed to specifically hybridize with human DNA, so our use of chimpanzee DNA required that probes non-specific to the chimpanzee genome, which could produce biased methylation measurements, be computationally filtered out and excluded from downstream analyses. This was accomplished using methods modified from Hernando-Herraez et al.[13]. Briefly, we used blastn to map the 866,837 50bp probes onto the chimpanzee genome (Assembly: Pan_tro_3.0, Accession: GCF_000001515.7) using an e-value threshold of e^{-10}. We only retained probes that successfully mapped to the genome, had only 1 unique BLAST hit, targeted CpG sites, had 0 mismatches in 5bp closest to and including the CpG site, and had 0-2 mismatches in 45bp not including the CpG site. This filtering retained 622,819 probes. Additionally, β values associated with cross-reactive probes, probes containing SNPs at the CpG site (either human or chimpanzee), probes detecting SNP information, probes detecting methylation at non-CpG sites, and probes targeting sites within the sex chromosomes were removed using the minfi package in R. This filtering retained a final set of 576,505 probes.

Bisulfite-PCR of chimpanzee cranial bones

Postmortem frontal skull bones from two different chimpanzees (chimpanzee 1 and chimpanzee 2) were provided by the Biomedical Primate Research Centre (BPRC, The Netherlands). Bones

were opportunistically collected during routine necropsy of these animals and stored at -80°C. Chimpanzee 3 and chimpanzee 4 samples were obtained from the chimpanzee cranial collection in the Department of Paleoanthropology in the Senckenberg Research Institute Frankfurt (DPSF) and Natural History Museum Frankfurt. These two chimpanzee specimens are owned by the Justus Liebig University Gießen.

Chimpanzee 1 and chimpanzee 2: For each sample, bone powder was obtained by crushing the bones with mortar and pestle. Approximately 100mg bone powder were used to extract DNA using the QIAamp DNA Investigator Kit (Qiagen) following manufacturer's instructions.

Chimpanzee 3 and chimpanzee 4: Cochlear bone powder was obtained by accessing the petrous bone from the cranial base[14]. DNA was extracted from about 50 mg of powder according to the protocol described by Dabney et al.[8], but adapted for the use of High Pure Nucleic Acid Large Volume columns (Roche) instead of the Zymo-Spin V column (Zymo Research) MinElute silica spin column (Qiagen) combination.

Genomic DNA was bisulfite converted with the EZ DNA Methylation – Lightning Kit (Zymo Research, D5030) according to the manufacturer's instructions. Specifically, each bone sample was bisulfite converted two times in parallel using 500ng as genomic DNA input for the conversion.

3µl of bisulfite treated DNA were amplified with the FastStart High Fidelity PCR System (Sigma, 03553400001) using the primers listed in Supplementary Table 1. PCR conditions were performed according to manufacturer's instructions and PCR products were visualized on a 1.5 % agarose gel. Prior to cloning, PCR products were purified with homemade SPRI beads (chimpanzee 1 and 2) and Gel/PCR DNA Mini Kit (RBC, YDF100, chimpanzee 3 and 4), and quantified with a NanoDrop 2000 spectrophotometer.

CloneJET PCR Cloning Kit (Thermo Scientific, K1231) was used to clone the purified PCR products into a pJET1.2/blunt Cloning Vector following the Blunt-End Cloning Protocol described in the manufacturer's instructions. 3µl (chimpanzee 1 and 2) and 3µl (chimpanzee 3 and 4) of each cloning reaction product were used for transformation of DH5α Competent Cells (Invitrogen, 18265017). Colonies were grown overnight on LB plates containing 100µg/ml ampicillin. Positive transformants were picked and grown overnight in liquid LB medium containing 100 µg/ml ampicillin. Subsequently, plasmid minipreps were purified with a QIAprep Miniprep Kit (Qiagen, chimpanzee 1 and 2), and RBC Miniprep Kit (YPD100, chimpanzee 3 and 4) according to manufacturer's instructions. Purified plasmids were quantified with a NanoDrop 2000 spectrophotometer and sequenced on an Applied Biosystems 3730xl Genetic Analyzer (Supplementary Figure 5a,b).

Reconstructing ancient DNA methylation maps

In a dedicated clean room at Harvard Medical School, powder was extracted from the root of a lower third molar of the Mesolithic La Braña 1 individual (5983-5747 calBCE (6980±50 BP, Beta-226472)), from which a non-UDG-treated library was previously sequenced to 3.5x coverage[15]. Two UDG-treated libraries from the same individual were later generated and enriched for approximately 1.2 million single targeted polymorphisms and sequenced to an average of 19.5x coverage at these position[16]. In this study, we carried out shotgun sequencing of one of the same UDG-treated libraries from this individual on a NextSeq500 instrument using 2 x 76bp paired end sequences. Following the mapping protocol described previously[16], we trimmed adapter sequences, only processed read pairs whose ends overlapped by at least 15 bp (allowing for one mismatch) so that we could confidently merge them, and then mapped to the human reference sequence hg19 using the command samse in BWA (v0.6.1). We removed

duplicated sequences by identifying sequences with the same start and stop position and orientation in the alignment, and picking the highest quality one. After restricting to sequences with a map quality of MAPQ ≥ 10, and sites with a minimum sequencing quality (≥20), we had an average coverage measured at the same set of approximately 1.2 million single nucleotide polymorphism targets of 23.0x. This data is available under GEO accession number: GSE96833, with raw reads deposited under SRA accession number: SRX3194436.

In a dedicated clean room at the University College Dublin, powder was extracted from the cochlear portion of the petrous bone of individual I1583 (archaeological ID L14-200) from the site of Barcın Höyük in the Yenişehir Plain of the Marmara Region of Northwest Turkey. The Neolithic individual came from a community that practiced farming, and was anthropologically determined to be a male aged 6-10 years at the time of death (the sex was confirmed genetically). The direct radiocarbon date was 6426-6236 calBCE (7460±50 BP, Poz-82231). In a dedicated clean room at Harvard Medical School, a UDG-treated library was prepared from this powder, which was previously enriched for about 1.2 million SNP targets, sequenced to 13.5x average coverage, and published in [16]. We shotgun sequenced the same library on nine lanes of a HiSeqX10 sequencing with 100bp paired reads. On data processing, we merged overlapping read pairs, trimmed Illumina sequencing adapters, and dropped read pairs that did not have sample barcodes (up to 1 mismatch) or cannot be unambiguously merged. We then aligned merged reads with BWA against human reference genome GRCh37 (hg19) plus decoy sequences, and combined all nine lanes of data and removed duplicate molecules, achieving an average of 24.3x coverage evaluated on the 1.2 million targets. This data is available under GEO accession number: GSE96833, with raw reads deposited under SRA accession number: SRX3194436.

The reconstruction procedure

Reconstruction of DNA methylation maps was performed on the genomes of the following individuals: Ust'-Ishim[3], Loschbour[17], Stuttgart[17], La Braña 1, I1583, and the Vindija Neanderthal[2], as well as on the previously published Altai Neanderthal and the Denisovan (Supplementary Data 1). The Vindija Neanderthal reads were downloaded from the Max Planck Institute for Evolutionary Anthropology website: http://cdna.eva.mpg.de/neandertal/Vindija/bam/. Only the UDG-treated portion of the genome (B8744) was used. Additional UDG-treated ancient human full genomes have been published to date; however, these were sequenced to a relatively low coverage (<5x), and thus, only crude methylation maps could be reconstructed from them. C→T ratio was computed for every CpG position along the hg19 (GRCh37) human genome assembly, for each of the samples[1].

In order to exclude from the analyses positions that potentially represent pre-mortem C→T mutations rather than post-mortem deamination, the following filters were applied: (i) Positions where the sum of A and G reads was greater than the sum of C and T reads were excluded. (ii) For genomes that were produced using single-stranded libraries (i.e., Ust'-Ishim, Altai Neanderthal, Denisovan, Vindija Neanderthal and ~1/3 of the Loschbour library), positions where the G→A ratio on the opposite strand was greater than 1/(average single strand coverage) were excluded. This fraction represents a threshold of one sequencing error allowed per position. For Loschbour, this was performed only on the fraction of reads that came from the single stranded library. (iii) For all genomes, positions with a C→T ratio > 0.25 were discarded. For the Vindija Neanderthal, this threshold was raised to 0.5, due to its relatively low coverage (~7x). (iv) Finally, a maximum coverage threshold of 100 reads was used to filter out regions that are suspected to be PCR duplicates.

In all genomes, excluding Vindija, a fixed sliding window of 25 CpGs was used for smoothing of the C→T ratio. This allowed for an unbiased scanning of differentially methylated regions (DMRs) that is not affected by the size of the window. Due to its relatively low coverage, we extended the sliding window used on the Vindija genome to 50 CpGs. This extended window is not expected to introduce a bias, as this genome was not used for DMR detection, but only for subsequent filtering that was applied equally to all genomes (see later).

C→T ratio was translated to methylation percentage using linear transformation determined from two points: zero C→T ratio was set to the value 0% methylation, and mean C→T ratio in completely methylated (100% methylation) CpG positions in modern human bone reference (hereinafter μ_{100}) was set to the value 100% methylation. Positions where C→T ratio > μ_{100} were set to 100% methylation. For genomes that were extracted from bones, the modern Bone 2 WGBS map, which is the one with the higher coverage between the two WGBS modern bone maps, was used to determine μ_{100}. For genomes that were extracted from teeth, there was no available modern reference methylation map, and therefore, we transformed the C→T ratio into methylation percentage based on the assumption that the genome-wide mean methylation is similar to bone tissue. Thus, the genome-wide mean C→T ratio represents 75% methylation, which is the genome-wide mean of measured methylation in the Bone 2 reference map. This was accomplished by setting μ_{100} to 1.33 x mean genome-wide C→T ratio.

DMR detection

The DMR detection algorithm is comprised of five main steps. We hereby provide an overview of the algorithm followed by a detailed description of each step. The overall goal of this pipeline is to detect differential methylation, assign it to the lineage on which it arose and filter out within-lineage variation.

Step 1: Two-way comparisons. To avoid artifacts that could potentially be introduced by comparing DNA methylation maps that were produced using different technologies, our core analysis relied on the comparison of the three reconstructed maps of the Altai Neanderthal, Denisovan, and Ust'-Ishim. Each of the samples was compared to the other two in a pair-wise manner, as a raw C→T ratio map against a reconstructed methylation map, and vice versa. This reciprocal comparison insured that the reconstruction process does not introduce biases to one of the groups. The minimum methylation difference threshold was set to 50%, spanning >50 CpGs.

Step 2: Three-way comparisons. This step classifies to which of the three hominins the DMR should be attributed. This step is done by overlapping the three lists of DMRs found in Step 1. For example, a DMR that is detected between the Neanderthal and Ust'-Ishim and also between the Denisovan and Ust'-Ishim is considered specific to Ust'-Ishim.

Step 3: FDR filtering. Various factors could introduce noise to the reconstruction process, including the stochasticity of the deamination process, the use of a sliding window, and variations in read depth within a sample. We ran simulations that mimic the post-mortem degradation processes of ancient DNA, then reconstructed methylation maps from the simulated deamination maps and finally compared them to the original map and identified DMRs. Any differences in methylation levels between the simulated map and the original reference map stem from noise. Thus, running the same DMR-detection algorithm on the simulated map vs. the

reference map, enables an estimation of the false discovery rate. We set the DMR-detection thresholds so that FDR < 0.05.

Step 4: Lineage assignment. The chimpanzee methylation maps were used to polarize the DMRs. For each DMR, methylation levels in the chimpanzee were compared to those of the three hominin groups. For example, if methylation levels in the chimpanzee samples clustered with the archaic humans, the DMR was assigned to the AMH lineage.

Step 5: Within-lineage variability filtering. To determine whether a DMR represents an individual within a group, or is shared by the entire group, we used a total of 67 AMH, archaic and chimpanzee methylation maps. We used a conservative approach where DMRs in which methylation levels in one group overlap (even partially) the methylation levels in another group were discarded. As 59 out of the 67 maps belong to AMHs, our ability to filter out variation within this group was better, resulting in fewer DMRs along this lineage. Several various measures were used to ascertain that a DMR along a lineage does not represent a sex-, bone-, age-, technology or disease-specific DMR.

DMR-detection algorithm

We developed an algorithm specifically designed to identify DMRs between a deamination map and a full methylome reference. Let i enumerate the CpG positions in the genome. In the deamination map, let t_i be the number of T's at the C position + the number of A's in the opposite strand at the G position, i.e., it counts the total number of T's that appear in a position that is originally C, in the context of a CpG dinucleotide. We similarly use c_i to count the total number of C's that appear in a position that is originally C, in the context of a CpG dinucleotide. The C→T ratio is defined as t_i/n_i, where $n_i = c_i + t_i$. Let φ_i and ψ_i (both between zero and one) be the methylation of this position in the reference genome and in the reconstructed one,

respectively. If we denote by π the deamination rate, assumed to be constant throughout the genome, and if we assume that deamination of C into T is a binomial process with probability of success $\pi\psi_i$, we get

$$t_i \sim B(n_i, \pi\psi_i). \tag{1}$$

Our null hypothesis is that the ith CpG is not part of a DMR, namely that $\psi_i = \varphi_i$. The alternative hypothesis states that this CpG is part of a DMR. The definition of this statement is that $|\psi_i - \varphi_i| \geq \Delta$, where Δ is some pre-specified threshold. In other words, under the alternative hypothesis we get that $\psi_i \geq \varphi_i + \Delta$ if the site has low methylation in the reference genome, and $\psi_i \leq \varphi_i - \Delta$ if it has high methylation in the reference genome.

Per-site statistic

Let us start with the first option, testing whether $\psi_i \geq \varphi_i + \Delta$ when φ_i is low. A log-likelihood-ratio statistic would be

$$\ell_i^+ = \ln \frac{\Pr\big(t_i \big| n_i, \pi(\varphi_i + \Delta)\big)}{\Pr(t_i | n_i, \pi\phi_i)} = t_i \left[\ln\left(1 + \frac{\Delta}{\varphi_i}\right) - \ln \frac{1 - \pi(\varphi_i + \Delta)}{1 - \pi\varphi_i}\right] + n_i \ln \frac{1 - \pi(\varphi_i + \Delta)}{1 - \pi\varphi_i}.$$

Similarly, we can test whether $\psi_i \leq \varphi_i - \Delta$ when φ_i is high using the log-likelihood-ratio statistic

$$\ell_i^- = \ln \frac{\Pr\big(t_i \big| n_i, \pi(\varphi_i - \Delta)\big)}{\Pr(t_i | n_i, \pi\phi_i)} = t_i \left[\ln\left(1 - \frac{\Delta}{\varphi_i}\right) - \ln \frac{1 - \pi(\varphi_i - \Delta)}{1 - \pi\varphi_i}\right] + n_i \ln \frac{1 - \pi(\varphi_i - \Delta)}{1 - \pi\varphi_i}.$$

We used the value $\Delta = 0.5$ for all samples. The value of π, the deamination rate, was estimated using the overall C→T ratio in CpG positions whose methylation level is 1 in the modern human Bone 2 WGBS methylation map, after exclusion of putative pre-mortem substitutions, as described in the reconstruction procedure section (Supplementary Data 1).

Detecting DMRs

The statistics ℓ_i^+ and ℓ_i^- quantify how strongly the estimated methylation in position i deviates from φ_i. Next, we use these values to identify DMRs using the cumulative-sum procedure explained below. The process is repeated twice: on the statistic ℓ_i^+ to identify DMRs where the sample has elevated methylation with respect to the reference, and on the statistic ℓ_i^- to identify DMRs where the sample has reduced methylation with respect to the reference.

For convenience, we explain the cumulative-sum procedure in the context of ℓ_i^+, but an essentially identical procedure is used for ℓ_i^-. We define a new vector Q^+ by the recursion

$$Q_0^+ = 0, \quad Q_i^+ = \max(Q_{i-1}^+ + \ell_i^+, 0).$$

Under the null hypothesis, ℓ_i^+ has a negative expectation which produces a negative drift that keeps Q^+ at zero, or close to zero, levels. Under the alternative hypothesis the expectation is positive, hence the drift over a DMR is positive, leading to an elevation in the values of Q^+. Therefore, our next step is to find all intervals $[a, b]$ such that $Q_{a-1}^+ = 0$, $Q_{b+1}^+ = 0$, and $Q_i^+ > 0$ for $a \leq i \leq b$. Let Q_m^+ be the maximum value of Q^+ in this interval, where m is the position of the maximum. Then, the interval $[a, m]$ would be called a putative DMR.

The statistics ℓ_i^+ and ℓ_i^- are affected by the number of observed cytosine reads, and thus have higher power to detect hypermethylation (i.e., larger number of cytosine reads) vs. hypomethylation (Supplementary Figure 3).

Filtering DMRs

Of course, Q^+ may increase locally due to randomness, and thus a putative DMR may not reflect a true DMR. To filter out such intervals, we used two strategies. First, we applied a set of filters to assure that the putative DMRs have reasonable biological properties. Second, we cleaned the remaining putative DMRs by applying a false discovery rate (FDR) procedure. In the first

strategy, we applied two filters: (i) Putative DMRs that harbor less than 50 CpG positions, thus are shorter than twice the smoothing window size, were removed. (ii) To avoid situations where two consecutive CpG sites whose genomic locations are remote appear on the same DMR, we modify the vector Q_i^+ as follows. Let $d_{i,j}$ be the distance along the genome (in nucleotides) between CpG sites i and j. Then, for every site i such that $d_{i,i-1} > \delta$ we set $Q_i^+ = 0$. We used $\delta = 1000$ nt for all samples.

To further remove putative DMRs that are unlikely to reflect true DMRs, we eliminated all DMRs where $Q_m^+ < Q_T^+$. Here, Q_m^+ is the maximum value of Q^+ in the interval as defined earlier, and Q_T^+ is a threshold determined using a false discovery rate (FDR) procedure, see the Filtering out noise section below.

Testing the algorithm

To verify that the approach above results in a low number of false positives, we applied the procedure to deamination maps, when compared to themselves in the form of reconstructed methylomes. As expected, we obtained a negligible number of DMRs, ranging between 0.4% and 1% of the number of DMRs detected between the humans.

Two-way DMR detection

In order to avoid artifacts that could potentially be introduced by comparing DNA methylation maps that were produced using different technologies, our core analysis relied on the comparison of the three reconstructed maps of the Altai Neanderthal, Denisovan, and Ust'-Ishim. These are all high-resolution maps that were derived from genomes sequenced to high coverage (Supplementary Data 1). In particular, the Ust'-Ishim methylome is of exceptional quality due to its high coverage and deamination rate (Supplementary Data 1). Also, going through the same

post-mortem degradation processes, the Ust'-Ishim cellular composition is likely to be similar to that of the Neanderthal and Denisovan.

In order for a deamination map to serve as a reference in the comparison, we have transformed its C→T ratio values into methylation values (see The reconstruction procedure section above). To remove potential bias that could be introduced through the comparison of a reconstructed methylation map to a deamination map, we ran each two-way comparison twice: once with the methylation map of sample 1 against the deamination map of sample 2, and once with the deamination map of sample 1 against the methylation map of sample 2 (Supplementary Figure 3). Therefore, the comparison of three genomes required a total of six two-way comparisons: Ust'-Ishim versus an Altai Neanderthal reference, Ust'-Ishim versus a Denisovan reference, Altai Neanderthal versus an Ust'-Ishim reference, Altai Neanderthal versus a Denisovan reference, Denisovan versus Ust'-Ishim reference, and Denisovan versus Altai Neanderthal reference. Because the DNA of these three individuals was extracted from both sexes, the DMR-detection algorithm was only applied to autosomes.

Three-way DMR detection

In order to identify DMRs where one group of humans (hereinafter, hominin 1) differs from the other two human groups (hereinafter, hominin 2 and hominin 3), we set out to find those DMRs that were detected both between hominin 1 and 2, and between hominin 1 and 3. To this end, we compare the two lists (hominin 1 vs. hominin 2 and hominin 1 vs. hominin 3) and look for overlapping DMRs[1]. An overlapping DMR exists when a DMR from one list partially (or fully) overlaps a DMR from the second list. Only the overlapping portion of the two DMRs from the two-lists was taken.

Filtering out noise

There are different factors that potentially introduce noise into the reconstruction process. These include the stochasticity of the deamination process, the use of a sliding window to smooth the C→T signal, and variations in read depth. In order to account for these factors and estimate noise levels, we ran simulations that mimic the post-mortem degradation processes of ancient DNA, then reconstructed methylation maps from the simulated deamination maps and finally compared them to the original map and identified DMRs.

The simulation process starts with a methylation map, where the measured or reconstructed methylation at position i is ψ_i and is assumed the true methylation. Given that n_i is the coverage at this position, we use the binomial distribution (1) to randomly draw t_i – the number of C's that had become T's through deamination. The resulting t_i's were then used to compute the C→T ratios for each position, smoothed and filtered using the same sliding window and thresholds used in the original analysis, and linearly transformed to methylation percentages as explained above (hereinafter, simulated methylation map, Supplementary Figure 2a). Any differences in methylation levels between the simulated map and the original reference map stem from noise. Thus, running the same DMR-detection algorithm described above on the simulated map vs. the reference map, enables an estimation of the false discovery rate. We ran these simulations 100 times for each of the three genomes (Altai Neanderthal, Denisovan, Ust'-Ishim) and determined the values of the Q_T^+ and Q_T^- thresholds (see the Filtering DMRs section above) such that the mean number of DMRs that are detected in the simulations is < 0.05 the number of real DMRs detected (i.e., FDR < 0.05).

DMRs separating chimpanzees and humans

To identify DMRs that separate chimpanzees from all human groups (both modern and archaic), we first compared the chimpanzee WGBS bone methylome to each of two present-day WGBS maps (those of Bone1 and Bone2). This was done by scanning the chimpanzee map using a sliding window of 25 CpGs, in intervals of one CpG position. In each window, we counted the number of methylated and unmethylated reads in each sample, and computed a P-value using Fisher's Exact test. We then computed FDR-adjusted P-values for each window, and discarded windows with FDR > 0.05 or where the mean methylation difference (Δ) was below 0.5. We then merged overlapping windows. This left 8,040 DMRs between the chimpanzee and Bone1, and 12,666 DMRs between the chimpanzee and Bone2. Next, we intersected the two lists to identify DMRs where the chimpanzee differs from the both present-day samples. This left 6,417 DMRs. Lastly, we compared the chimpanzee methylation levels to all other human samples (modern and archaic) and filtered out DMRs where the chimpanzee is found within the range of methylation levels observed in humans. To do so, we followed the procedure described in the Removing DMRs with high within-group variability section below. This resulted in 2,031 DMRs that separate chimpanzees and humans.

Determining the lineages where DMRs originated

DMRs where Ust'-Ishim differs from the Neanderthal and the Denisovan could either arose on the AMH branch, or in the ancestor of Neanderthals and Denisovans. In order to allocate the DMRs to the branch in which the change occurred, we used the chimpanzee DNA methylation data.

First, we used the chimpanzee bone WGBS map. We defined the distance of a DMR in hominin H to chimpanzee as the mean absolute difference in methylation, $d_{H,C} = \sum_{i \in DMR} |\psi_i^H - \psi_i^C|$.

Here, ψ_i^H is the reconstructed methylation at the i'th CpG in hominin H, and ψ_i^C is the measured methylation in the same site in the chimpanzee. For Ust'-Ishim-specific DMRs, we used the following procedure: (i) If both archaic humans were closer to the chimpanzee, the DMR was placed on the AMH branch. (ii) If Ust'-Ishim was closer than both archaic humans to the chimpanzee, the DMR was placed on the branch of the ancestor of Neanderthals and Denisovans. (iii) Otherwise, the DMR was discarded. Out of 5,111 Ust'-Ishim-specific DMRs, we could place 1,729 DMRs on the AMH branch and 1,255 on the branch of the ancestor of Neanderthals and Denisovans. 1,807 Ust'-Ishim-specific DMRs were discarded due to inconclusive lineage assignment, and 320 had no data in the chimpanzee WGBS map. For Neanderthal-specific DMRs, we discarded all DMRs where Ust'-Ishim and the Denisovan were not found to be closer to the chimpanzee than the Neanderthal. Out of 3,107 Neanderthal-specific DMRs, 693 were placed on the Neanderthal branch, 2,202 were deemed inconclusive and were discarded, and 212 had no data in the chimpanzee WGBS map. Similarly, we discarded Denisovan-specific DMRs where Ust'-Ishim and Altai Neanderthal were not found to be closer to the chimpanzee than the Denisovan. Out of 1,461 Denisovan-specific DMRs, 499 were placed on the Denisovan branch, 855 were deemed inconclusive, and for 107 we had no data in the chimpanzee WGBS map.

We next developed a second, stricter, scheme by also using the chimpanzee 850K DNA methylation arrays datasets. As the probes cover just part of the CpGs in a DMR, we need to adjust the DMR methylation level in order to allow a meaningful comparison of 850K methylation data to full methylation maps. If we mark by j the CpGs in a DMR that are covered by 850K methylation array (which is a subset of all the CpGs in this DMR), and mark their total number by $J = \sum_{j \in DMR} 1$, then the methylation in the DMR as measured by the array is $m = 1/J \cdot \sum_{j \in DMR} \psi_j^{\text{array}}$, where ψ_j^{array} is the methylation level measured at position j in the array. Let

$m_I = \sum_{i \in DMR} \psi_i^{\text{WGBS}}$ be the methylation of this DMR as computed from the full methylation map, where ψ_i^{WGBS} is the methylation level measured at position i in the full map. Let $m_J = \sum_{j \in DMR} \psi_j^{\text{WGBS}}$ be the methylation as computed from the full methylation map when limited only to positions j. Then, we correct the array methylation value m to:

$$m' = \min\left(m \cdot \frac{m_I}{m_J}, 1\right). \qquad (2)$$

This procedure was applied to DMRs covered by at least one probe (~65% of DMRs). For the remaining ~35% of DMRs, we only used the WGBS chimpanzee methylome. This approach was used in parallel with filtering DMRs using the modern human 450K arrays (Supplementary Figure 3, see next section).

There are pros and cons to each of these approaches. Using more chimpanzee datasets allow for more informative process. However, 850K methylation array probes are distributed unevenly across the genome. Although most DMRs are covered by at least one probe (mean number of probes per DMR: 1.7, median: 1, maximum: 64), many are nonetheless not covered. AMH

On one hand, lineage assignment of DMRs for which we have array data is more robust and less prone to misclassification. On the other hand, DMRs with array data are more likely to be filtered out, as there is more power to detect variability. This could potentially alter the genomic distribution of DMRs. Therefore, we use both approaches throughout the paper. In analyses where it is important to maintain an unbiased distribution of DMRs we only use the chimpanzee WGBS map for polarization, and AMH bone WGBS maps for filtering (see next chapter), whereas in analyses where it is more important to minimize variability, or where we look at specific DMRs, we use the stricter approach. The chimpanzee RRBS data was adjusted using the same technique. However, it was not used for lineage assignment, but rather only as a source for

additional information on DMRs. This is because this protocol particularly targets unmethylated CpGs, and is therefore too biased for lineage assignment.

Removing DMRs with high within-group variability

Our three-way DMR detection algorithm above produces a list of DMRs where one of the three hominins (Ust'-Ishim, Altai Neanderthal or Denisovan) is significantly different from the other two. However, such DMRs could stem from variability within any of the groups, and in such cases cannot be regarded as truly differentiating between the human groups. Some variability may be removed during the process described above (see the Determining the lineages where DMRs originate section), but even DMRs whose origin can be assigned to a particular lineage do not necessarily represent fixed methylation changes. To filter out regions that are variable within any of the human groups, or across all of them, we used two approaches. First, we used the two modern human WGBS maps, and the I1583 reconstructed skull methylation map. DMRs where the Neanderthal or Denisovan methylation levels were found within the range of modern human methylation (i.e., Ust'-Ishim, the two WGBS maps and I1583) were discarded. This left 1,530 out of 1,729 Ust'-Ishim-derived DMRs (hereinafter, full AMH-derived DMRs), 1,230 out of 1,255 DMRs where the Neanderthal and Denisovan are both derived, 692 out of 693 full Neanderthal-derived DMRs, and 496 out of 499 Denisovan-derived DMRs.

The second approach adds to this the 52 450K methylation array samples, as well as the three reconstructed methylation maps from teeth (i.e., Loschbour, Stuttgart and La Braña 1). As described above, using also methylation probes for filtering DMRs provides more power, but can also introduce biases. Thus, this filtering was used for most analyses, except those where unbiased genomic distribution of DMRs is critical. Probe methylation data was corrected as described in equation (2). Within AMH- and archaic-derived DMRs, a DMR was deemed fixed

if the Neanderthal and the Denisovan methylation levels both fell outside the range of methylation across all modern human samples (reconstructed, WGBS and 450K maps). Similarly, within Neanderthal- and Denisovan-derived DMRs, a DMR was deemed fixed if the respective hominin fell outside the range of methylation across all modern human samples and the other archaic hominin. This approach yielded 873 AMH-derived DMRs (hereinafter referred to as AMH-derived DMRs), 939 archaic-derived DMRs, 570 Neanderthal-derived DMRs, and 443 Denisovan-derived DMRs.

The limited number of archaic human methylation maps introduces asymmetry in our ability to determine the level of fixation of DMRs along different lineages. Whereas we used dozens of AMH skeletal samples, we have just a few archaic samples. This provides us with the ability to better estimate the distribution of methylation values within each DMR in AMH, and thus to determine how significantly methylation values in other samples deviate from it. To enhance our ability to estimate variability within archaic human lineages, we added to the analysis the reconstructed methylation map of the Vindija Neanderthal. The USER-treated portion of this genome (the portion amenable for methylation reconstruction) was sequenced[2] to a depth of 7x. Therefore, the methylation map that could be reconstructed from this individual has a considerably lower resolution compared to the other reconstructed maps used in this study (coverage 19x to 52x). Nevertheless, due to the reduced ability to detect variability along the archaic human linages, we employed this map for additional variability filtering along these lineages. DMRs where the Vindija Neanderthal clustered with the other hominins, and not with the Altai Neanderthal (or not with either of the archaic humans in the archaic-derived DMRs) were discarded. The number of DMRs mentioned throughout this chapter already includes this filtering.

A general concern in working with DNA methylation data is that DMRs that are specific to one group do not necessarily represent an evolutionary change, but rather reflect a characteristic such as technology used to measure methylation, tissue, sex, disease or age that is shared by individuals in this group and not by others. We take two complementary approaches to ascertain that the DMRs we report are not driven by these factors: (a) for the top DMGs, we match the samples for the above factors and test whether the hypermethylation of AMHs is still observed. To this end, we compared Ust'-Ishim (adult femur with no known diseases, methylation map produced using our reconstruction method) to the Vindija Neanderthal (adult femur with no known diseases, methylation map produced using our reconstruction method), and we also compared 52 modern human samples (adult femora, methylation array maps) to four chimpanzee samples (adult femora, methylation array maps). In all cases, AMHs show significant hypermethylation compared to the matched samples (Supplementary Figure 3c,d, see the Methylation in AMH, chimpanzee and Neanderthal femora chapter for additional information). (b) throughout the pipeline, we take only DMRs where one human group clusters completely outside the other groups regardless of tissue, sex, disease, age or technology. Thus, these factors are unlikely to drive the reported methylation changes. This approach is particularly useful in AMH-derived DMRs, where each group of samples (i.e., AMH samples vs. archaic and chimpanzee samples) include both males and females, juveniles and adults, and they come from femora, ribs, tibia, skulls, and teeth. Thus, it is unlikely that the DMRs that differentiate these groups reflect variability that stems from these parameters[18] (Fig. 1a-c). Archaic-derived DMRs and Neanderthal-derived DMRs are also unlikely to reflect differences in the above parameters, as in these DMRs, the Vindija Neanderthal sample (adult, femur bone) is clustered with the Altai Neanderthal sample (juvenile/adult, phalanx), and not with AMHs, where most samples are from

femora of adult females. Denisovan-derived DMRs, on the other hand, are more likely to stem from age or bone type differences than other types of DMRs. This is because the Denisovan sample is the only finger bone, and it comes from a child (6-13.5 years) (Supplementary Data 1). Thus, we cannot rule out the possibility that some of the Denisovan-derived DMRs reflect finger-specific, rather than lineage-specific methylation patterns. These DMRs could also possibly reflect age-specific differences, but this is less likely, as the AMH I1583 sample[16] and the chimpanzee 850K samples are the same age group as the Denisovan (Denisovan: 6-13.5 years old, I1583: 6-10, chimpanzees: 10-13) but show different methylation patterns than the Denisovan (Supplementary Data 1, Fig. 1).

Note that we do not generally expect the number of DMRs along a lineage to be proportional to the length of the lineage, as this number is determined by several factors. First, the statistical power to detect DMRs depends on coverage and deamination levels. Thus, our ability to detect DMRs was lowest in the Denisovan, and highest in Ust'-Ishim. Second, the ability to filter out within-population variability was substantially higher along the AMH lineage, to which most samples belong. While filtering out such variability, we also exclude variability that exists across both AMH and archaic populations. This filtering also discards genomic regions that are variable between sexes, bone types and regions where methylation patterns tend to be more stochastic. Variability that exists exclusively along the Neanderthal lineage was partially removed using the Vindija Neanderthal sample, which comes from a different bone (femur vs. phalanx) and age (adult vs. juvenile/adult). Along the Denisovan-lineage, on the other hand, such variability could not be filtered out using our array of samples (Fig. 1).

We also repeated the Gene ORGANizer analyses (see the Gene ORGANizer analysis section) after removal of 20 DMRs that overlap regions which were shown to change methylation during

osteogenic differentiation[19]. We show that the enrichment of voice-affecting genes holds, and thus, the differentiation state of cells in the samples is unlikely to explain the results we report.

Comparison to previous reports

We have previously reported that compared to present-day humans, the HOXD cluster of genes is significantly hypermethylated in the Neanderthal and Denisovan samples[1]. Using the current methylation maps, we show that this observation holds (Supplementary Figure 2b). Adding chimpanzee data, we see that similarly to AMHs, chimpanzee samples are also hypomethylated compared to archaic humans. This suggests that the hypermethylation arose along the archaic-human lineage. However, we find that the Ust'-Ishim individual is an outlier among modern humans, and that his methylation levels are closer to the Neanderthal than to modern humans, as was also shown by Hanghøj et al.[20]. The Neanderthal and Ust'-Ishim individuals are found >2 standard deviations from the mean observed methylation in modern humans. This suggests that although the Neanderthal is hypermethylated compared to almost all modern humans, she is not found completely outside modern human variation. The Denisovan, on the other hand, is found even further away, and significantly outside the other populations. Given this, the HOXD DMR was classified as Denisovan-derived (Supplementary Data 2). The Ust'-Ishim remains include a single femur, and to our knowledge, it was not compared morphologically to other humans. Thus, further analysis is needed in order to determine whether the hypermethylation of the Ust'-Ishim individual compared to other AMHs is manifested in morphological changes as well. Moreover, as this DMR is classified as Denisovan-derived, we cannot rule out the possibility that it is driven to some extent by age or bone type differences.

Compared to the previously reported DMRs[1], in this study we found four times as many AMH- and archaic-derived DMRs (2,805 full bone DMRs compared to 891) and roughly twice as many

Neanderthal- and Denisovan-derived DMRs (440 and 598 compared to 295 and 307 in the Denisovan and Neanderthal, respectively). The list of DMRs reported here cannot be directly compared to our previous list of DMRs because of several key differences in the analysis: (i) The previous study focused on DMRs that are invariable across tissues, whereas here we focused on DMRs in skeletal tissues. In the previous study, we were therefore able to extrapolate and find trends that extend beyond the skeletal system, such as neurological diseases. In this paper we focus on the skeletal system, hence the different appearance of the body map (Fig. 2b,c). (ii) the current study used stricter thresholds for DMR detection, including a minimum of 50 CpGs in each DMR (compared to 10 CpGs previously), and a requirement for physical overlap in the three-way DMR detection procedure. (iii) In this study, the AMH reference is a reconstructed ancient map, whereas in the previous study the AMH reference, as well as the other tissues used for filtering out noise, were mainly cultured cell lines with RRBS methylation maps.

When filtering DMRs along the lines of the previous study by taking only DMRs with low inter-tissue variability in humans (STD < 10%), we indeed observe similar trends. For example, when taking AMH-derived DMGs and analyzing their expression patterns using DAVID's tissue expression tools, we found that the brain is the most represented organ, with 51.5% of DMGs expressed in this organ (x1.28, FDR = 2.6×10^{-4}), and glial cells are the most over-represented cell type (x20.6, but FDR > 0.05, UP_TISSUE DB, Supplementary Data 3). In fact, the brain is the only significantly enriched organ in this analysis. Similarly, when analyzing the GNF DB, we found that the subthalamic nucleus is the most enriched body part (x1.60, FDR = 9.2×10^{-4}), followed by additional brain regions, such as the olfactory bulb (x1.54, FDR = 0.01), globus pallidus (x1.41, FDR = 0.04), and more (Supplementary Data 3). Similar enrichment patterns of the brain can be observed when analyzing expression patterns of all AMH-derived DMGs

(Supplementary Data 3). Finally, we also find that similarly to the previous report, these DMRs are linked to diseases more often (23.1% compared to the genome average of 10.8%, DAVID OMIM_DISEASE DB).

Computing correlation between methylation and expression

In order to identify regions where DNA methylation is tightly linked with expression levels, we scanned each DMR in overlapping windows of 25 CpGs (the window used for smoothing the deamination signal). In each window we computed Pearson's correlation between DNA methylation levels and expression levels of overlapping genes as well as the closest genes upstream and downstream genes, across 21 tissues[21]. For each DMR, we picked the window with the best correlation (in absolute value) and computed regression FDR-adjusted *P*-value. DMRs that overlap windows with FDR < 0.05 were considered to be regions where methylation levels are significantly correlated with expression levels. 90 such DMRs were found among the skeletal AMH-derived DMRs, 93 among the archaic human-derived DMRs, 40 among Neanderthal-derived DMRs, and 19 among Denisovan-derived DMRs.

As no expression data were available for Ust'-Ishim, Bone1 and Bone2, we approximated their *NFIX* expression level by taking the average of *NFIX* expression from three osteoblast RNA-seq datasets that were downloaded from GEO accession numbers GSE55282, GSE85761 and GSE78608. RNA-seq data for chondrocytes was downloaded from the ENCODE project, GEO accession number GSE78607 and plotted against measured methylation levels in primary chondrocytes (see the Human primary chondrocyte validation chapter). Notably, even though the expression and methylation data come from different individuals, plotting them against one another positions them only ~one standard deviation from the expression value predicted by the regression line (Fig. 5b). Future studies providing RNA expression levels for the laryngeal

skeleton and vocal folds might provide further information on the methylation-expression links of these genes.

Studying the function of DMGs

Gene ontology and expression analyses were conducted using Biological Process and UNIGENE expression tools in DAVID, using an FDR threshold of 0.05.

Gene ORGANizer analysis

Similarly to sequence mutations, changes in regulation are likely to be unequally distributed across different body systems, owing to negative and positive selection, as well as inherent traits of the genes affecting each organ. Thus, we turned to investigate which body parts are affected by the DMGs. To this end, we ran the lists of DMGs in Gene ORGANizer[22], which is a tool that links genes to the organs they affect, through known disease and normal phenotypes. Thus, it allows us to investigate directly the phenotypic function of genes, to identify their shared targets and to statistically test the significance of such enrichments. We ran the lists of DMGs in the ORGANize option using the default parameters (i.e., based on *confident* and *typical+non-typical* gene-phenotype associations).

When we ran the list of skeletal AMH-derived DMRs, we found 11 significantly enriched body parts, with the vocal folds and the larynx being the most enriched parts (x2.11 and x1.68, FDR = 0.017 and FDR = 0.046, respectively). Most other parts belonged to the face (teeth, forehead, lips, eyelid, maxilla, face, jaws), as well as the pelvis and nails (Fig. 2c,d, Supplementary Data 4). For archaic-derived DMGs, the lips, limbs, jaws, scapula, and spinal column were enriched (Supplementary Figure 4e, Supplementary Data 4). The Neanderthal-derived and Denisovan-derived DMG lists did not produce any significantly enriched organs, but the immune system was significantly depleted within Neanderthal-derived DMRs (x0.67, FDR = 0.040).

In order to examine whether such trends could arise randomly from the reconstruction method, we repeated the analysis on the previously described 100 simulations. We ran all simulated DMGs (4,153) in Gene ORGANizer and found that no enrichment was detected, neither for voice-related organs (vocal folds: x0.99, FDR = 0.731, larynx: x.1.02, FDR = 0.966, FDR = 0.966), nor for any other organ.

Validation of face and larynx enrichment in Gene ORGANizer

To test whether the enrichment of the face and larynx could be attributed to the fact that the analyses are based on skeletal tissues, we tested whether the proportion of genes related to the face, larynx, vocal folds and pelvis within AMH-derived skeleton-related DMGs is higher than expected by chance. Out of 100 skeleton-related DMGs, 31 genes are known to affect the voice, 34 affect the larynx, 87 affect the face, and 65 affect the pelvis, whereas genome-wide these proportions are significantly lower (14.2%, 20.2%, 70.0%, 52.4%, $P = 1.0 \times 10^{-5}$, $P = 1.3 \times 10^{-3}$, $P = 2.1 \times 10^{-3}$, P = 0.03, for vocal folds, larynx, face, and pelvis, respectively, hypergeometric test). For additional validation tests, see main text.

Genes associated with craniofacial features were taken from the GWAS-catalog (version 2019-04-21), using a threshold of $P < 10^{-8}$. The following features were used: Dental caries, Cleft palate, Facial morphology, Intracranial volume, Cleft palate (environmental tobacco smoke interaction), Cranial base width, Craniofacial macrosomia, Facial morphology (factor 1, breadth of lateral portion of upper face), Facial morphology (factor 10, width of nasal floor), Facial morphology (factor 11, projection of the nose), Facial morphology (factor 12, vertical position of sublabial sulcus relative to central midface), Facial morphology (factor 14, intercanthal width), Lower facial height, Nose morphology, Nose size, Tooth agenesis (maxillary third molar), Tooth agenesis (third molar), facial morphology traits (multivariate analysis), Lower

facial morphology traits (ordinal measurement), Lower facial morphology traits (quantitative measurement), Middle facial morphology traits (quantitative measurement), and Upper facial morphology traits (ordinal measurement). We then tested their overlap with DMGs. Genes associated with craniofacial features in the GWAS catalog significantly overlapped DMGs compared to the fraction expected by chance (5.17x, $P = 3.4 \times 10^{-4}$, hypergeometric test). As a control, we then tested how this 5-fold enrichment compares to non-craniofacial features. We used blood-related GWAS as a representative of general non-craniofacial GWAS. We extracted from the GWAS catalog 22 blood-related traits (the same number as extracted for craniofacial features), by taking the first 22 traits that appear in a search for the term *blood* and applying a threshold of $P < 10^{-8}$. We then used these genes as a background control for the craniofacial enrichment. We observed a 3.86x enrichment of DMGs with regard to craniofacial- vs. non-craniofacial-associated genes ($P = 0.01$, chi-square test).

Additionally, we conducted a permutation test on the list of 129 AMH-derived DMGs that are linked to organs on Gene ORGANizer, replacing those that are linked to the skeleton with randomly selected skeleton-related genes. We then ran the list in Gene ORGANizer and computed the enrichment. We repeated the process 100,000 times and found that the enrichment levels we observed within AMH-derived DMGs are significantly higher than expected by chance for the laryngeal and facial regions, but not for the pelvis ($P = 8.0 \times 10^{-5}$, $P = 3.6 \times 10^{-3}$, $P = 8.2 \times 10^{-4}$, and $P = 0.115$, for vocal folds, larynx, face and pelvis, respectively, permutation test, Supplementary Figure 4b-e).

Potentially, longer genes have higher probability to overlap DMRs. Indeed, DMGs tend to be longer (148 kb vs. 39 kb, $P = 9.9 \times 10^{-145}$, *t*-test). We thus checked the possibility that genes affecting the larynx and face tend to be longer than other genes, and are thus more likely to

contain DMRs. We found that length of genes could not be a factor explaining the enrichment within genes affecting the larynx, as these genes tend to be shorter than other genes in the genome (mean: 62.5 kb vs. 73.2 kb, $P = 0.001$, t-test). Genes affecting the face, on the other hand, tend to be longer than other genes (mean: 77.1 kb vs. 65.6 kb, $P = 4.6 \times 10^{-5}$, t-test). To examine if this factor may underlie the enrichment we observe, we repeated the analysis using only DMRs that are found within promoter regions (5 kb upstream to 1 kb downstream of TSS), thus eliminating the gene length factor. We found that the genes where such DMRs occur are still significantly associated with the face ($P = 0.036$, Fisher's exact test). We next repeated the promoter DMR analysis for all genes and compared the Gene ORGANizer enrichment levels in this analysis to the genome-wide analysis. We observed very similar levels of enrichment (2.02x, 1.67x, and 1.24x, for vocal folds, larynx, and face, respectively, albeit FDR values > 0.05 due to low statistical power). Importantly, AMH-derived DMGs also do not tend to be longer than DMGs on the other branches (148 kb vs. 147 kb, $P = 0.93$, t-test). Together, these analyses suggest that gene length does not affect the observed enrichment in genes affecting the face and larynx.

Additionally, to test whether cellular composition or differentiation state could bias the results, we ran Gene ORGANizer on the list of DMGs, following the removal of 20 DMRs that are found <10 kb from loci where methylation was shown to change during osteogenic differentiation[19]. We found that genes affecting the voice and face are still the most over-represented (2.13x, 1.71x, and 1.27x, FDR = 0.032, FDR = 0.049, and FDR = 0.040, for vocal folds, larynx, and face, respectively, Supplementary Data 4).

We also investigated the possibility that (for an unknown reason) the DMR-detection algorithm introduces positional biases that preferentially identify DMRs within genes affecting the voice or

face. To this end, we simulated stochastic deamination processes along the Ust'-Ishim, Altai Neanderthal, and Denisovan genomes, reconstructed methylation maps, and ran the DMR-detection algorithm on these maps. We repeated this process 100 times for each hominin and found no enrichment of any body part, including the face, vocal folds, or larynx (1.07x, 1.07x, and 1.04x, respectively, FDR = 0.88 for vocal folds, larynx, and face, permutation test). Perhaps most importantly, none of the other archaic branches shows enrichment of the larynx or the vocal folds. However, archaic-derived DMGs show over-representation of the jaws, as well as the lips, limbs, scapulae, and spinal column (Supplementary Figure 4e, Supplementary Data 4). In addition, DMRs that separate chimpanzees from all humans (archaic and modern, Supplementary Data 2) do not show over-representation of genes that affect the voice, larynx, or face, compatible with the notion that this trend emerged along the AMH lineage. We also sought to test whether the larynx and vocal folds, which we found to be significantly enriched only along the AMH lineage, are also enriched when compared to the other lineages. We ran a chi-squared test on the fraction of vocal folds- and larynx-affecting AMH-derived DMGs (25 and 29, respectively, out of a total of 120 organ-associated DMGs), compared to the corresponding fraction in the DMGs along all the other lineages (42 for vocal folds, 49 for larynx, out of a total of 275 organ-associated DMGs). We found that both the larynx and vocal folds are significantly enriched in AMHs by over 50% compared to the other lineages (1.57x for both, P = 0.0248 and P = 0.0169 for vocal folds and larynx, respectively, chi-squared test).

Furthermore, we added a human bone reduced representation bisulfite sequencing (RRBS) map, and produced a RRBS map from a chimpanzee infant unspecified long bone (Supplementary Data 1). RRBS methylation maps include information on only ~10% of CpG sites, and are biased towards unmethylated sites. Therefore, they were not included in the previous analyses.

However, we added them in this part as they originate from a chimpanzee infant and a present-day human that is of similar age to the Denisovan (Supplementary Data 1), allowing sampling from individuals that are younger than the rest. Repeating the Gene ORGANizer analysis after including these samples in the filtering process, we found that the face and larynx are the only significantly enriched skeletal regions, and the enrichment within voice-affecting genes becomes even more pronounced (2.33x, FDR = 7.9 x 10^{-3}, Supplementary Data 4).

We also examined if pleiotropy could underlie the observed enrichments. To a large extent, the statistical tests behind Gene ORGANizer inherently account for pleiotropy[22], hence the conclusion that the most significant shared effect of the AMH-derived DMGs is in shaping vocal and facial anatomy is valid regardless of pleiotropy. Nevertheless, we tested this possibility more directly, estimating the pleiotropy of each gene by counting the number of different Human Phenotype Ontology (HPO) terms that are associated with it across the entire body[23]. We found that DMGs do not tend to be more pleiotropic than the rest of the genome ($P = 0.17$, *t*-test), nor do differentially methylated voice- and face-affecting genes tend to be more pleiotropic than other DMGs ($P = 0.19$ and $P = 0.27$, respectively, *t*-test).

Next, we tested whether the process of within-lineage removal of variable DMRs and the differential number of samples along each lineage biases the Gene ORGANizer enrichment analysis. To do so, we analyzed the pre-filtering DMRs along each lineage. We detect very similar trends to the post-filtering analysis, with the laryngeal and facial regions being the most significantly enriched within AMH-derived DMRs (1.58x, 1.44x and 1.21x-1.31x for the vocal folds, larynx and different facial regions, respectively, FDR < 0.05), and for archaic-derived DMRs, we detect no enrichment of the laryngeal region (FDR = 0.16 and FDR = 0.43 for the vocal folds and larynx, respectively), and the most enriched regions are the face, limbs, and

urethra. With the exception of the urethra, these results are very similar to the results reported for the filtered DMRs, suggesting that the process of within-lineage removal of variable DMRs and the differential number of samples along each lineage does not bias the enrichment results. Overall, we observe that AMH-derived DMGs across all 60 AMH samples are found outside archaic human variability, regardless of bone type, disease state, age, or sex, and that chimpanzee methylation levels in these DMGs cluster closer to archaic humans than to AMHs, suggesting that these factors are unlikely to underlie the observed trends.

Finally, we tested whether the filtering process in itself might underlie the observed trends. To this end, we re-ran the entire pipeline on Neanderthal- and Denisovan-derived DMGs, while applying to them all the filters as if they were Ust'-Ishim DMGs. This resulted in substantially fewer loci (89 for the Neanderthal and 50 for the Denisovan), which limits statistical power, but can still be used to examine whether there are any trends of enrichment similar to those observed in AMHs. We found no evidence that the filtering process could drive the enrichment of the vocal or facial areas: within Neanderthal-derived loci, filtered as if they were Ust'-Ishim-derived, we found that the vocal folds were ranked only 18th, with a non-significant enrichment of 1.27x (FDR = 0.815, compared to an enrichment of 2.11x within AMH-derived DMGs). The larynx was ranked 76th and showed a non-significant depletion of 0.87x (FDR = 0.783), and the face was ranked 31st, with a non-significant enrichment of 1.09x (FDR = 0.815). Within Denisovan-derived loci, filtered as if they were Ust'-Ishim-derived, none of the loci were linked to the vocal folds nor to the larynx (FDR = 0.535 and FDR = 0.834, respectively), and the face was ranked 30th (1.29x, FDR = 0.535, Supplementary Data 4). This test suggests that the filtering process in itself is very unlikely to underlie the enrichment of the vocal and facial parts within AMH-derived DMGs.

Next, we applied the Neanderthal/Denisovan filters to the Ust'-Ishim-derived loci. This resulted in 792 loci. We found that the vocal folds remained the most enriched body part (1.76x, FDR = 0.032), the larynx was marginally significant (1.53x, FDR = 0.0502), and the facial region was significantly enriched too (e.g., cheek and chin ranked 2nd, 3rd within significantly enriched body parts, 1.66x and 1.63x, FDR = 0.031 and FDR = 0.013, respectively, Supplementary Data 4). Importantly, we do not rule out the option that extensive regulatory changes in genes related to vocal and facial anatomy might have occurred along the Neanderthal and Denisovan lineages as well. Indeed, as we report in Supplementary Figure 4e, parts of the face are enriched within Archaic-derived DMGs. However, we currently see no substantial evidence supporting this. Importantly, the link between genetic alterations and phenotypes related to the voice is complex. Some brain-related disorders (i.e., clinical disorders that affect the brain) result in alterations to the voice, the mechanism in which is very difficult to pin down. Although the mechanism leading to voice alterations (either in its pitch, timbre, volume or range) in some of the genes we report is unknown, many of the disorders are skeletal, suggesting the mechanism is related to anatomical changes to the vocal tract. Such changes could also affect more primary functions of the larynx, such as swallowing and breathing. However, the enrichment we observe in Gene ORGANizer shows these genes were also shown to drive vocal alterations in the disorders they underlie[22,23]. Voice and speech alterations were also shown to be driven by cultural, dietary and behavioral changes affecting bite configuration[24]. Here too, these factors are unlikely to underlie the vocal alterations in the genes we report, as individuals from the same family as the individual with the disorder, who do not carry the dysfunctional allele, were not reported to present any vocal phenotypes.

The larynx is an organ which is primarily involved in breathing and swallowing in mammals. In humans, the larynx is also used to produce complex speech, but not every change to the larynx necessarily affects speech. Despite these additional functions, the genes reported by Gene ORGANizer and HPO were specifically associated with voice alterations, directly or indirectly, suggesting that although they could have additional effects, their effect on the voice is their most shared function.

Overlap with enhancer regions

To further test whether the AMH-derived DMRs overlap skeletal regulatory regions, we examined the previously reported 403,968 human loci, where an enrichment of the active enhancer mark H3K27ac was detected in developing human limbs (E33, E41, E44, and E47)[25]. Each DMR was allocated a random genomic position in its original chromosome, while keeping its original length and matching the distribution of GC-content and CpG density between the original and permutated lists. GC-content and CpG density matching was done by matching a 10-bin histogram of the original and permutated lists. This was repeated for 10,000 iterations. We found that AMH-derived DMRs overlap limb H3K27ac-enriched regions ~2x more often than expected by chance (610 overlapping DMRs, compared to 312.4±21.7, $P < 10^{-4}$, permutation test).

SOX9 upstream putative enhancer coordinates used in Fig. 4b were taken from [26–30].

Computing the density of changes along the genome

We computed the density of derived CpG positions along the genome in two ways. First, we used a 100 kb window centered in the middle of each DMR and computed the fraction of CpGs in that window which are differentially methylated (i.e., are found within a DMR). Second, for the chromosome density plots, we did not center the window around each DMR, but rather used

a non-overlapping sliding 100 kb window starting at position 1 and running the length of the chromosome.

NFIX, COL2A1, SOX9, ACAN and XYLT1 phenotypes

The vocal tract and larynx affecting genes presented in this paper show involvement in laryngeal cartilage and soft tissue phenotypic variation. Clinical phenotypes can be of high severity, with substantial impacts on normal breathing functions, to the point where the cause of death is due to respiratory distress. *SOX9* and *NFIX* are often associated with laryngomalacia[22,23] (Supplementary Data 5), a collapse of the larynx due to malformation of the laryngeal cartilaginous

framework and/or malformed connective tissues, particularly during inhalation. Patients with mutations in *COL2A1* often show backwards displacement of the tongue base[22,23]. Less severe phenotypes of the reported genes include variation of voice quality in the form of pitch variation (high in patients suffering from *XYLT1* mutations) and sometimes hoarseness of the voice (reported for some patients with mutations of *ACAN*, Supplementary Data 5)[22,23]. Whether this is due to variation of the vocal tract and laryngeal anatomy influenced by the *ACAN* mutation or due to a scaled down vocal tract size in the case of the *XYLT1* mutation which also causes primordial dwarfism is not yet clear.

NFIX phenotypes

Skeletal phenotypes that are associated with the Marshall-Smith syndrome were extracted from the Human Phenotype Ontology (HPO)[23]. Non-directional phenotypes (e.g., irregular dentition) and phenotypes that are expressed in both directions (e.g., tall stature and short stature) were removed.

Mutations in *NFIX* have also been linked to the Sotos syndrome. However, *NFIX* is not the only gene that was linked to this syndrome; mutations in *NSD1* were also shown to drive similar phenotypes[31]. Therefore, it is less relevant in assessing the functional consequences of general shifts in the activity levels of *NFIX*. Nevertheless, it is noteworthy that in the Sotos syndrome too, most symptoms are a mirror image of the Neanderthal phenotype (e.g., prominent chin and high forehead).

Comparing gene expression between AMH and mouse

93 appendicular skeleton samples were used to compare expression levels of *NFIX*, *SOX9*, *ACAN* and *COL2A1* in human and mouse: 1. Five Human expression array data of iliac bones[32], downloaded from ArrayExpress accession number E-MEXP-2219. 2. 84 Human expression array of iliac bones, downloaded from ArrayExpress accession number E-MEXP-1618. 3. Three Mouse expression array data of femur and tibia bones, downloaded from ArrayExpress accession number E-GEOD-61146. 4. One Mouse RNA-seq of a tibia bone, downloaded from supplementary data. Expression values were converted to percentiles, according to each gene expression level compared to the rest of the genome across each sample (Fig. 5c).

Methylation in AMH, chimpanzee and Neanderthal femora

To check whether the AMH hypermethylation of *SOX9, ACAN, COL2A1, XYLT1* and *NFIX* could be a result of variability between bone types, we compared the four chimpanzee femur 850K methylation arrays to the 52 present-day femur 450K methylation arrays. We took probes within AMH-derived DMRs that appear on both arrays. We found that these genes are consistently hypermethylated in AMHs (P = 1.6x10^{-7}, *t*-test), with 38 probes showing >5% hypermethylation in AMH, whereas only eight probes show such hypermethylation in chimpanzees (Supplementary Figure 5d). Therefore, even when comparing methylation from the

same bone, same sex, same developmental stage, measured by the same technology, and across the same positions, AMH show consistent hypermethylation across all of these DMGs. Similarly, when comparing the DMRs in *SOX9*, *ACAN*, *COL2A1*, *XYLT1*, and *NFIX* between the Ust'-Ishim and Vindija Neanderthal samples, the Vindija Neanderthal sample is consistently hypomethylated compared to the Ust'-Ishim individual ($P = 1.2 \times 10^{-5}$, Supplementary Figure 5c, *t*-test). Both of these samples were extracted from femora of adult individuals, and methylation was reconstructed using the same technology. This suggests that the hypermethylation of AMHs compared to Neanderthals is unlikely to be driven by age or bone type, and rather reflects evolutionary shifts.

Scanning *SOX9* for mutations altering NFI binding motifs

To examine whether the changes in regulation of *SOX9* could possibly be explained by changes in the binding sites of NFI proteins, we searched for the NFI motif along the gene body and the 350 kb upstream region of *SOX9*. We looked for NFI motifs that exist in the genomes of the Altai and Vindija Neanderthal, as well as in the Denisovan, but were abolished in AMHs. We did not find any evidence of such substitutions.

Comparison to divergent traits between Neanderthals and AMHs

To further investigate potential phenotypic consequences of the DMGs we report, we probed the HPO database[23] and compared these HPO phenotypes to known morphological differences between Neanderthals and modern humans[33]. To compile a list of traits in which Neanderthals and AMHs differ, we reviewed key sources that surveyed Neanderthal morphology summarized in Aiello, L. & Dean[34]. We identified traits in which Neanderthals are found completely outside AMH variation, as well as traits where one group is significantly different from the other, but the distribution of observed measurements partially overlap. Non-directional traits (i.e., traits that

could not be described on scales such as higher/lower, accelerated/delayed etc.) were not included, as could not be paralleled with HPO phenotypes. The compiled list included 107 phenotypes, 75 of which have at least one equivalent HPO phenotype (4.8 on average). For example, the HPO phenotype Taurodontia (HP:0000679) was linked to the trait Taurodontia, and the following HPO phenotypes were linked to the trait Rounded and robust rib shafts: *Broad ribs* (HP:0000885), *Hypoplasia of first ribs* (HP:0006657), *Short ribs* (HP:0000773), *Thickened cortex of long bones* (HP:0000935), *Thickened ribs* (HP:0000900), *Thin ribs* (HP:0000883), *Thoracic hypoplasia* (HP:0005257). For each skeleton-affecting phenotype, we determined whether it matches a known morphological difference between Neanderthals and AMHs[33]. For example, *Hypoplastic ilia* (HPO ID: HP:0000946) was marked as divergent because in the Neanderthal the iliac bones are considerably enlarged compared to AMHs[34]. We then counted for each gene (whether DMG or not) the fraction of its associated HPO phenotypes that are divergent between Neanderthals and AMHs. We found that four of the top five most differentially methylated skeletal genes (*XYLT1*, *NFIX*, *ACAN*, and *COL2A1*) are in the top 100 genes with the highest fraction of divergent traits between Neanderthals and AMHs (out of a total of 1,789 skeleton-related genes). In fact, *COL2A1*, which is the top ranked DMR (Supplementary Data 2), is also the gene that is overall associated with the highest number of derived traits (63) (Supplementary Data 7). This suggests that these extensive methylation changes are possibly linked to phenotypic divergence between archaic and AMHs.

References

1. Gokhman, D. *et al.* Reconstructing the DNA methylation maps of the Neandertal and the Denisovan. *Science (80-.).* **344**, (2014).
2. Prüfer, K. *et al.* A high-coverage Neandertal genome from Vindija Cave in Croatia. *Science (80-.).* **358**, 655–658 (2017).
3. Fu, Q. *et al.* Genome sequence of a 45,000-year-old modern human from western Siberia. *Nature* **514**, 445–9 (2014).

4. Horvath, S. *et al.* The cerebellum ages slowly according to the epigenetic clock. *Aging (Albany. NY).* **7**, 294–306 (2015).
5. Kulis, M. *et al.* Whole-genome fingerprint of the DNA methylome during human B cell differentiation. *Nat. Genet.* **47**, 746–756 (2015).
6. Marco-Sola, S., Sammeth, M., Guigó, R. & Ribeca, P. The GEM mapper: fast, accurate and versatile alignment by filtration. *Nat. Methods* **9**, 1185–1188 (2012).
7. Hansen, K. D., Langmead, B. & Irizarry, R. A. BSmooth: from whole genome bisulfite sequencing reads to differentially methylated regions. *Genome Biol.* (2012). doi:10.1186/gb-2012-13-10-R83
8. Dabney, J. *et al.* Complete mitochondrial genome sequence of a Middle Pleistocene cave bear reconstructed from ultrashort DNA fragments. *Proc. Natl. Acad. Sci. U. S. A.* **110**, 15758–63 (2013).
9. Rohland, N. & Hofreiter, M. Ancient DNA extraction from bones and teeth. *Nat. Protoc.* **2**, 1756–1762 (2007).
10. Boyle, P. *et al.* Gel-free multiplexed reduced representation bisulfite sequencing for large-scale DNA methylation profiling. *Genome Biol.* **13**, R92 (2012).
11. Barnett, R. & Larson, G. A phenol-chloroform protocol for extracting DNA from ancient samples. *Methods Mol. Biol.* **840**, 13–19 (2012).
12. Fortin, J. P., Triche, T. J. & Hansen, K. D. Preprocessing, normalization and integration of the Illumina HumanMethylationEPIC array with minfi. *Bioinformatics* (2017). doi:10.1093/bioinformatics/btw691
13. Hernando-Herraez, I. *et al.* Dynamics of DNA Methylation in Recent Human and Great Ape Evolution. *PLOS Genet* **9**, e1003763 (2013).
14. Sirak, K. A. *et al.* A minimally-invasive method for sampling human petrous bones from the cranial base for ancient DNA analysis. *Biotechniques* **62**, 283–289 (2017).
15. Olalde, I. *et al.* Derived immune and ancestral pigmentation alleles in a 7,000-year-old Mesolithic European. *Nature* **507**, 225–228 (2014).
16. Mathieson, I. *et al.* Genome-wide patterns of selection in 230 ancient Eurasians. *Nature* **528**, 499–503 (2015).
17. Lazaridis, I. *et al.* Ancient human genomes suggest three ancestral populations for present-day Europeans. *Nature* **513**, 409–413 (2014).
18. Gokhman, D., Meshorer, E. & Carmel, L. Epigenetics: It's Getting Old. Past Meets Future in Paleoepigenetics. *Trends Ecol. Evol.* **31**, (2016).
19. Håkelien, A. M. *et al.* The regulatory landscape of osteogenic differentiation. *Stem Cells* **32**, 2780–2793 (2014).
20. Hanghøj, K. *et al.* Fast, Accurate and Automatic Ancient Nucleosome and Methylation Maps with epiPALEOMIX. msw184 (2016). doi:10.1093/molbev/msw184
21. Roadmap Epigenomics Consortium *et al.* Integrative analysis of 111 reference human epigenomes. *Nature* **518**, 317–329 (2015).
22. Gokhman, D. *et al.* Gene ORGANizer: Linking genes to the organs they affect. *Nucleic Acids Res.* **45**, W138–W145 (2017).
23. K??hler, S. *et al.* The Human Phenotype Ontology project: Linking molecular biology and disease through phenotype data. *Nucleic Acids Res.* **42**, (2014).
24. Blasi, D. E. *et al.* Human sound systems are shaped by post-Neolithic changes in bite configuration. *Science (80-.).* (2019). doi:10.1126/science.aav3218
25. Guo, M. *et al.* Epigenetic profiling of growth plate chondrocytes sheds insight into

regulatory genetic variation influencing height. *Elife* **6**, (2017).
26. Yao, B. *et al.* The SOX9 upstream region prone to chromosomal aberrations causing campomelic dysplasia contains multiple cartilage enhancers. *Nucleic Acids Res.* **43**, 5394–5408 (2015).
27. Bagheri-Fam, S. *et al.* Long-range upstream and downstream enhancers control distinct subsets of the complex spatiotemporal Sox9 expression pattern. *Dev. Biol.* **291**, 382–397 (2006).
28. Sekido, R. & Lovell-Badge, R. Sex determination involves synergistic action of SRY and SF1 on a specific Sox9 enhancer. *Nature* **453**, 930–934 (2008).
29. Prescott, S. L. *et al.* Enhancer Divergence and cis-Regulatory Evolution in the Human and Chimp Neural Crest. *Cell* **163**, 68–84 (2015).
30. Wilderman, A., VanOudenhove, J., Kron, J., Noonan, J. P. & Cotney, J. High-Resolution Epigenomic Atlas of Human Embryonic Craniofacial Development. *Cell Rep.* **23**, 1581–1597 (2018).
31. Malan, V. *et al.* Distinct effects of allelic NFIX mutations on nonsense-mediated mRNA decay engender either a sotos-like or a Marshall-Smith Syndrome. *Am. J. Hum. Genet.* **87**, 189–198 (2010).
32. Varanasi, S. S. *et al.* Skeletal site-related variation in human trabecular bone transcriptome and signaling. *PLoS One* **5**, (2010).
33. Gokhman, D. *et al.* Reconstructing Denisovan Anatomy Using DNA Methylation Maps. *Cell* **179**, 180-192.e10 (2019).
34. Aiello, L. & Dean, C. *An Introduction to Human Evolutionary Anatomy*. (Elsevier, 2002).

Supplementary Figures

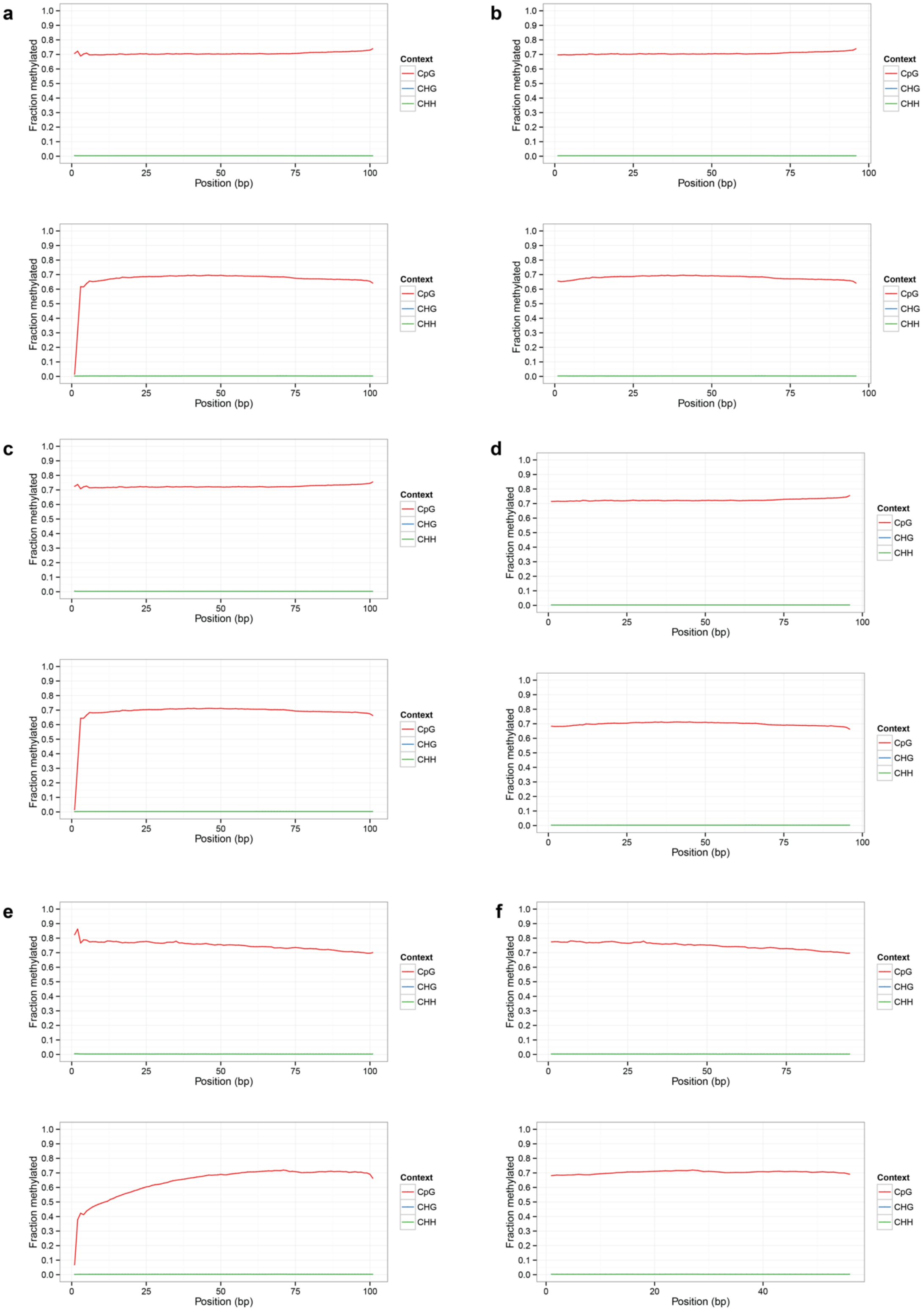

Supplementary Figure 1. **M-bias plots along reads in bone sample 1 and sample 2**. **a**. Pre-filtering methylation along read1 and read2 in the autosomes of bone 1. **b**. Post-filtering methylation along read1 and read2 in the autosomes of bone 1. **c**. Pre-filtering methylation along read1 and read2 in the autosomes of bone 2. **d**. Post-filtering methylation along read1 and read2 in the autosomes of bone 2. **e**. Pre-filtering methylation along read1 and read2 in the chimpanzee rib sample autosomes. **f**. Post-filtering methylation along read1 and read2 in the chimpanzee rib sample autosomes.

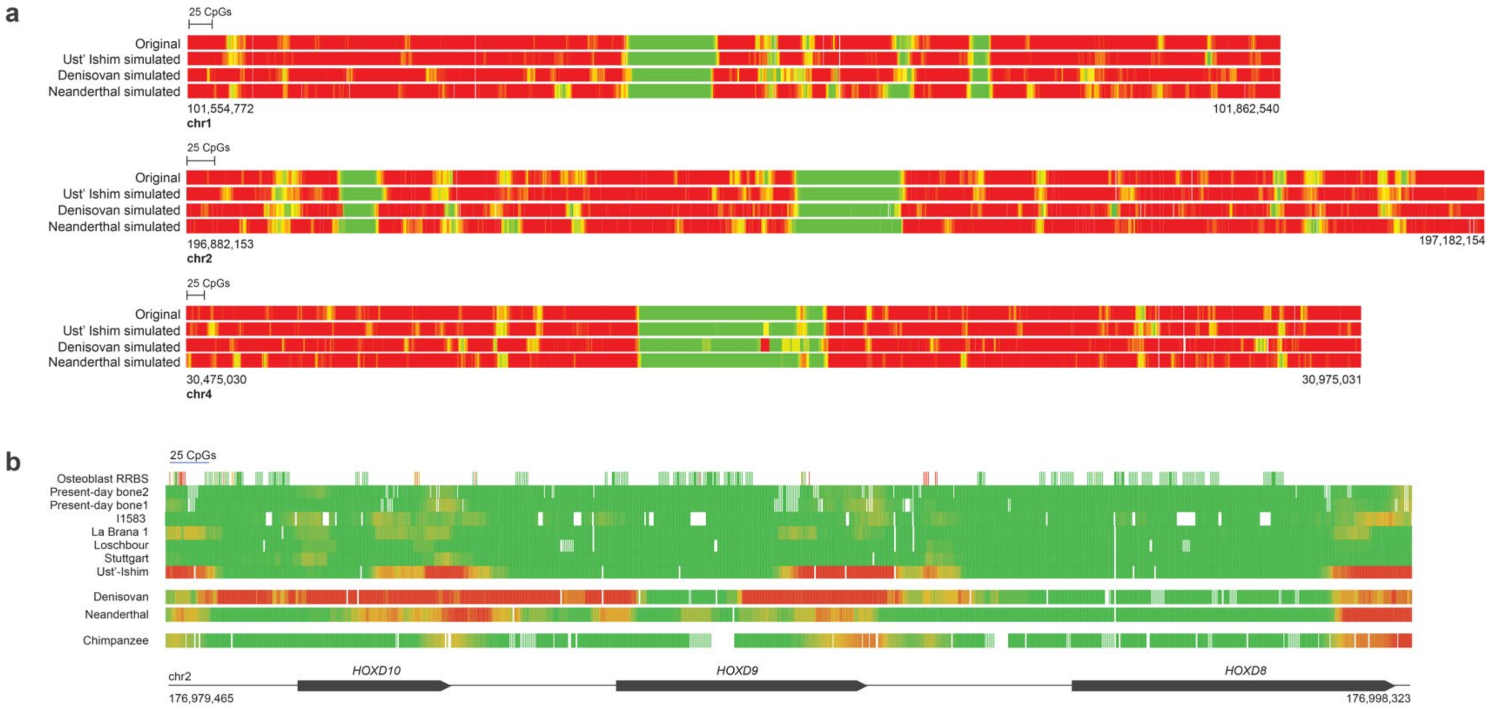

Supplementary Figure 2. Simulations of deamination and reconstruction, and comparison to previous reports. a. Simulations of cytosine deamination, followed by reconstruction reproduce DNA methylation maps. Deamination was simulated for each position based on its methylation level, read coverage and the observed rate of deamination in each hominin. Then,

DNA methylation maps were reconstructed and matched against the original map. The number of DMRs found were used as an estimate of false discovery rate. Three exemplary regions are presented, where methylation levels are color-coded from green (unmethylated) to red (methylated). **b.** The HOXD cluster is hypermethylated in archaic humans, and in the Ust'-Ishim individual. Methylation levels are color-coded from green (unmethylated) to red (methylated). The top eight bars show ancient and present-day AMH samples, the lower three show the Denisovan, Neanderthal and chimpanzee. The promoter region of *HOXD9* is hypermethylated in the Neanderthal and the Denisovan, but not in AMHs. The 3' ends of the three genes are hypermethylated in the Neanderthal, Denisovan, Ust'-Ishim and chimpanzee, but not in other AMH samples. The promoter of HOXD10 is methylated only in the Denisovan.

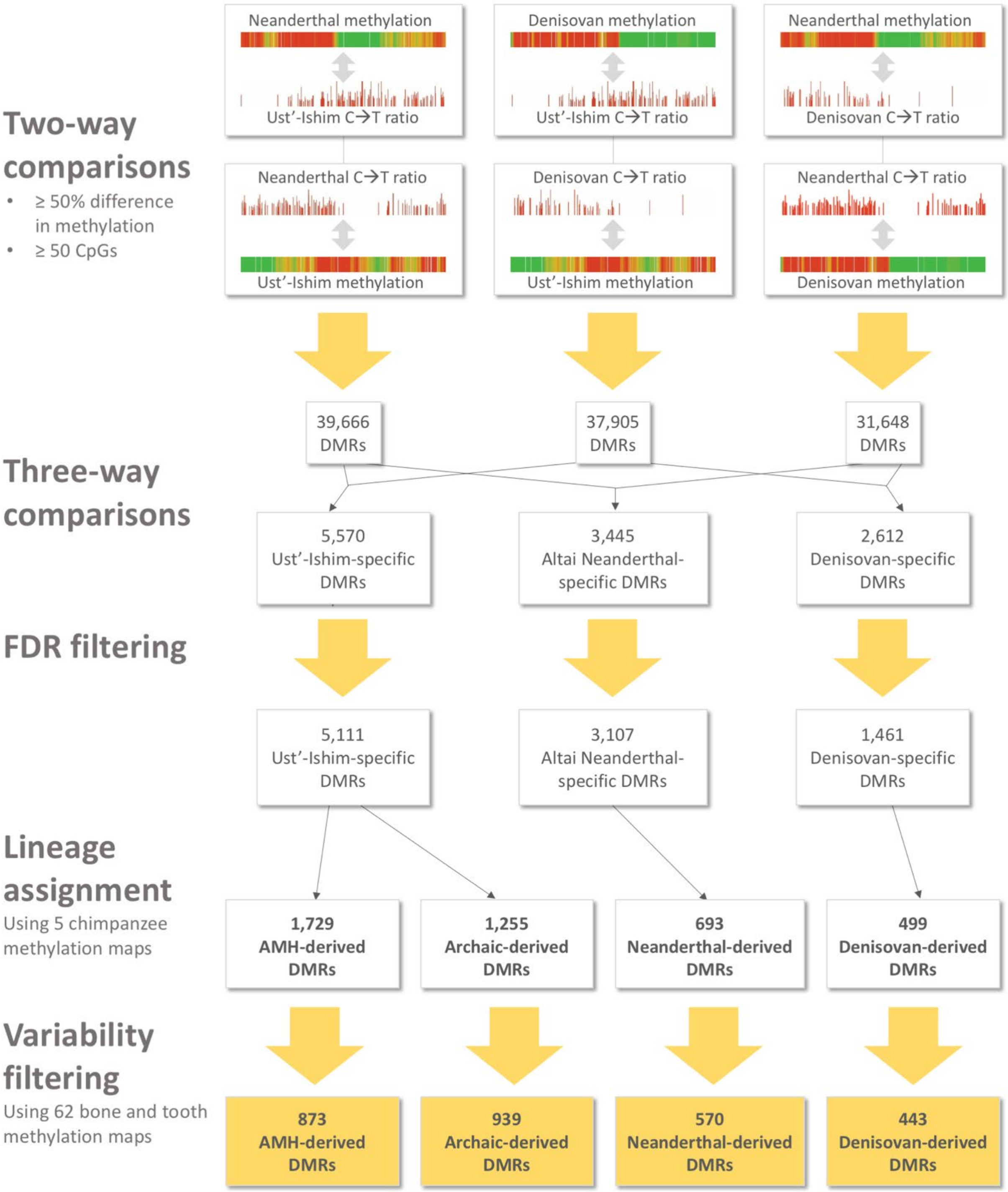

Supplementary Figure 3. DMR-detection flowchart. At the core of the process are six two-way (pairwise) comparisons between the Altai Neanderthal, Denisovan, and Ust'-Ishim

individuals. In each two-way comparison, a C→T deamination signal of one hominin was compared to the reconstructed methylation map of the other hominin. This resulted in three lists of pairwise DMRs, that were then intersected to identify hominin-specific DMRs, defined as DMRs that appear in two of the lists. False discovery rates were controlled by running 100 simulations for each hominin, each simulating the processes of deamination, methylation reconstruction, and DMR-detection. Only DMRs that passed FDR thresholds of < 0.05 were kept. To discard non-evolutionary DMRs we used 62 skeletal methylation maps, and kept only loci whose methylation levels differed in one lineage, regardless of age, bone type, disease or sex. Finally, five chimpanzee methylation maps were used to assign the lineage in which each DMR likely emerged.

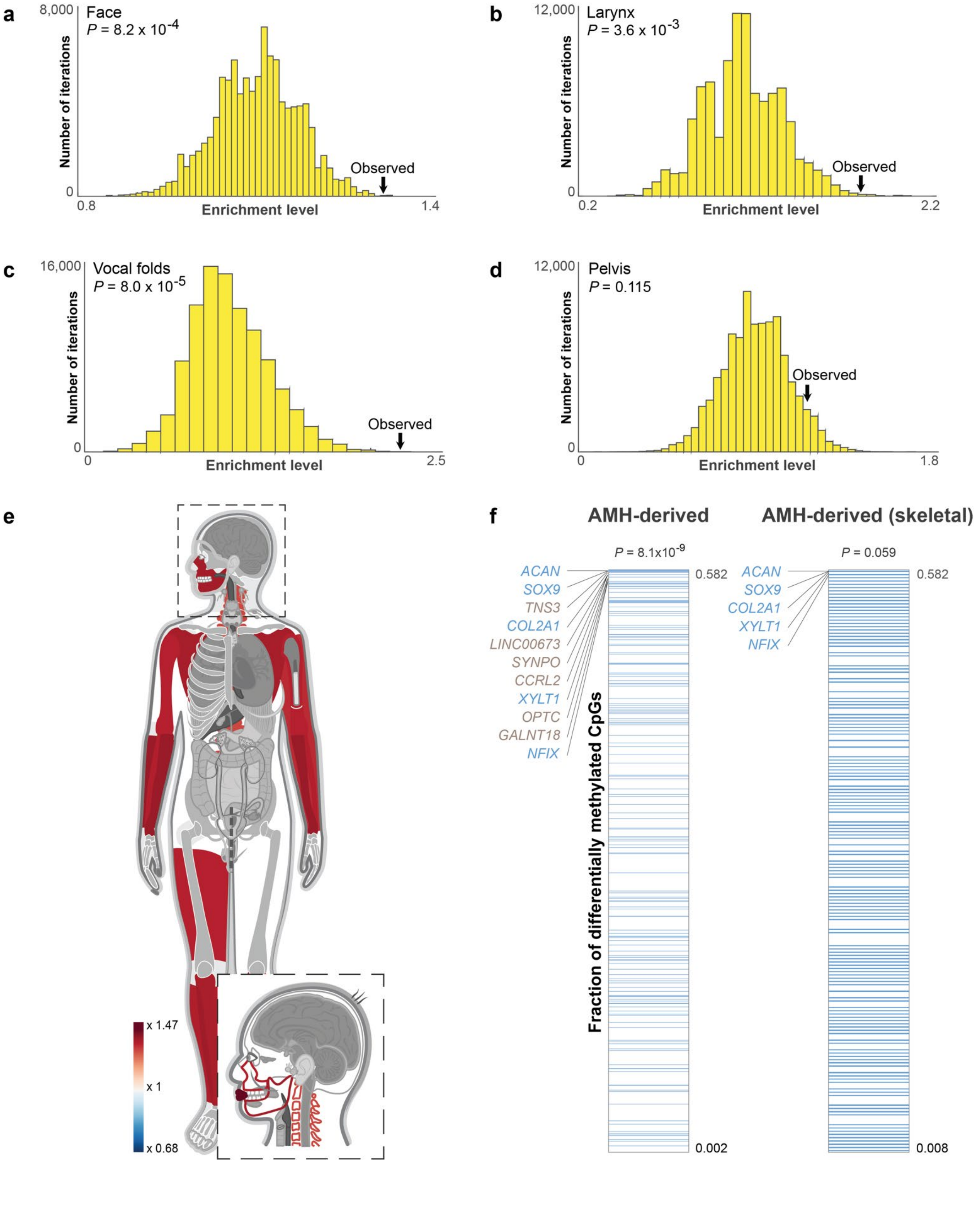
a
8,000
Face
P = 8.2 x 10^{-4}
Number of iterations
Observed
0
0.8
Enrichment level
1.4
b
12,000
Larynx
P = 3.6 x 10^{-3}
Number of iterations
Observed
0
0.2
Enrichment level
2.2
c
16,000
Vocal folds
P = 8.0 x 10^{-5}
Number of iterations
Observed
0
0
Enrichment level
2.5
d
12,000
Pelvis
P = 0.115
Number of iterations
Observed
0
0
Enrichment level
1.8
e
x 1.47
x 1
x 0.68
f
AMH-derived
AMH-derived (skeletal)
P = 8.1x10^{-9}
P = 0.059
ACAN
SOX9
TNS3
COL2A1
LINC00673
SYNPO
CCRL2
XYLT1
OPTC
GALNT18
NFIX
Fraction of differentially methylated CpGs
0.582
0.002
ACAN
SOX9
COL2A1
XYLT1
NFIX
0.582
0.008

Supplementary Figure 4. The face and larynx are enriched within AMH-derived DMGs compared to genes affecting the skeleton, and compared to archaic-derived DMGs. a-d. The distribution of enrichment levels in 100,000 randomized lists of genes, where non-skeletal AMH-derived DMGs were unchanged, whereas skeleton-related DMGs were replaced with random skeleton-related genes. Observed enrichment levels are significantly higher than expected in the face, larynx, and vocal folds. **e.** A heat map representing the level of enrichment of each anatomical part within archaic-derived DMGs. Genes affecting the lips, limbs, jaws, scapula, and spinal column are the most enriched within archaic-derived DMRs. Only body parts that are significantly enriched (FDR < 0.05) are colored. **f.** The number of AMH-derived CpGs per 100 kb centered around the middle of each DMR. DMRs were ranked according to the fraction of derived CpG positions within them. DMRs in genes affecting the face are marked with blue lines. In AMHs, DMRs in face-affecting genes tend to be ranked significantly higher. Although only ~2% of genes in the genome are known to affect lower and midfacial projection, three of the top five AMH-derived DMRs, and all top five AMH-derived skeleton-affecting DMRs are within genes known to affect facial projection. *t*-test *P*-value is shown.

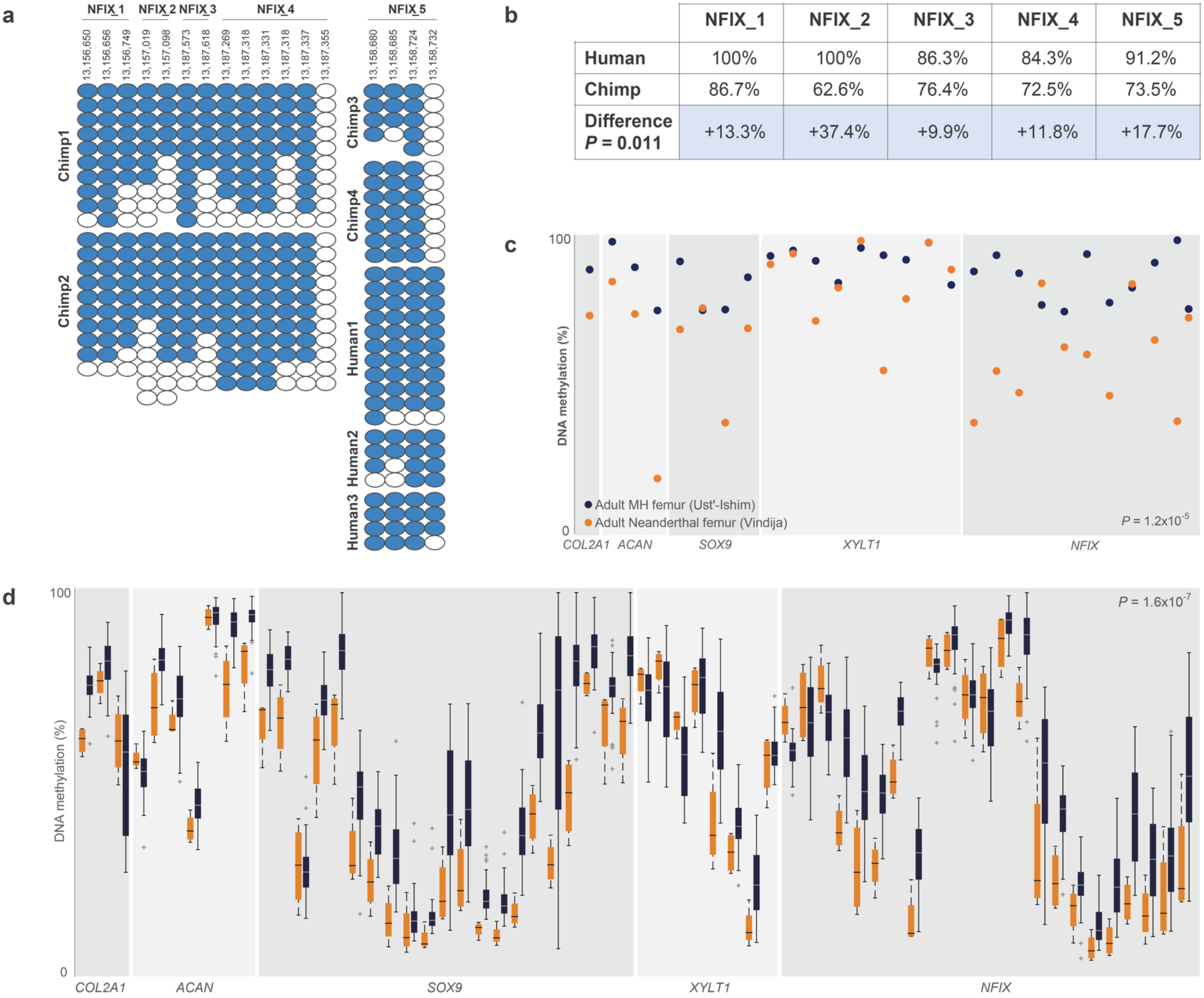

Supplementary Figure 5. AMHs are hypermethylated compared to Neanderthal and chimpanzee bone samples, even when age and bone type are considered. a,b. Bisulfite-PCR in human and chimpanzee crania of five regions within the two *NFIX* DMRs, showing hypermethylation of NFIX in AMHs (P = 0.01, t-test). Each column represents a CpG position, with each circle representing either methylated (blue) or unmethylated (white) measurements. Human hg19 coordinates are shown for each CpG position. Chimpanzee methylation in regions

1-4 was compared with the human I1583 cranium. Region 5 was compared with I1583 and three additional present-day human crania, presented in the figure. Summarized results are presented in the table. **c.** *COL2A1, ACAN, SOX9,* and *NFIX* are hypermethylated in Ust'-Ishim (blue) compared to the Vindija Neanderthal (orange). Circles represent mean methylation levels in AMH-derived DMRs. Both samples were extracted from femora of adults, and methylation was reconstructed using the same method. The DMRs presented include also those that were analyzed in the density analyses. The hypermethylation of these genes in AMHs is unlikely to be attributed to age or bone type. *t*-test *P*-value is shown. **d.** *COL2A1, ACAN, SOX9,* and *NFIX* are hypermethylated in AMH femora compared to chimpanzee femora. Each pair of box plots represents methylation levels across 52 AMH femora (blue) and four chimpanzee femora (orange) in a single probe of methylation array. Central line shows mean, box borders show 25th and 75th percentiles, whiskers extend to the most extreme data points not considered outliers, crosses show outliers. When comparing methylation in the same bone, measured by the same technology, and across the same positions, AMHs show almost consistent hypermethylation compared to chimpanzee. The probes presented include also probes within DMRs that were analyzed in the density analyses. *t*-test *P*-value is shown.

Supplementary Tables

Extraction	Mg used	ug/mL	Volume in uL	Total ug
Extraction 1	50	1.28	115	0.1472
Extraction 2	100	2.51	115	0.2886
Extraction 3	100	1.8	115	0.2162
Blank control 1	n/a	<0.010 ug/mL	115	0
Blank control 2	n/a	<0.010 ug/mL	115	0

Supplementary Table 1. DNA extractions from chimpanzee femurs.

Animal ID	Species	Sex	Age (yr)	Array ID	Position
4X0523	chimpanzee	male	10.20	200670680029	R02C01
4X0406	chimpanzee	male	10.26	200665030025	R07C01
4X0387	chimpanzee	male	13.46	200705360064	R07C01
4-0191	chimpanzee	unknown	unknown	200705860055	R05C01

Supplementary Table 2. Chimpanzee methylation array samples.

Region	Forward primer	Reverse primer
NFIX_1	GGGATTGGAGAATTTGTTTTTGT	AAAAAATATCAACCCTTCCC
NFIX_2	TTTTTGTTGATTAAAGGGGT	ATACCCTTAAACACAAAACT
NFIX_3	TTTTTATTGGGAAGGGTTAA	CCTCCCAAAAAAAAAAAAAC
NFIX_4	TTTTTTGAGATTAGGTAGGGAA	TCACACCCACAATAAAAATAAAC
NFIX_5 (chimp)	GGAAGATAGGTTTTTTTTTTAT	AAAAATCAACCTTTACCTTCATC
NFIX_5 (human)	GGAAGATAGGTTTTTTTTTTGT	AAAAATCAACCTTTACCTTCATC

Supplementary Table 3. Primers used for Bisulfite-PCR.
