# Supplementary material for: Differential DNA methylation of vocal and facial anatomy genes in modern humans
Source: Nat Commun. 2020 Mar 4;11:1189. doi: 10.1038/s41467-020-15020-6 (PMC7055320; doi:10.1038/s41467-020-15020-6)
Supplement: Supplementary file 11 — Reporting Summary [file 41467_2020_15020_MOESM11_ESM.pdf]

nature research

Corresponding author(s): *Double-blind peer review submissions: write DBPR and your manuscript number here instead of author names.*

Last updated by author(s): *YYYY-MM-DD*

Reporting Summary

Nature Research wishes to improve the reproducibility of the work that we publish. This form provides structure for consistency and transparency in reporting. For further information on Nature Research policies, seeAuthors & Referees and theEditorial Policy Checklist .

Statistics

For all statistical analyses, confirm that the following items are present in the figure legend, table legend, main text, or Methods section.

n/a	Confirmed	
☐	☒	The exact sample size (*n*) for each experimental group/condition, given as a discrete number and unit of measurement
☒	☐	A statement on whether measurements were taken from distinct samples or whether the same sample was measured repeatedly
☐	☒	The statistical test(s) used AND whether they are one- or two-sided<br>*Only common tests should be described solely by name; describe more complex techniques in the Methods section.*
☒	☐	A description of all covariates tested
☐	☒	A description of any assumptions or corrections, such as tests of normality and adjustment for multiple comparisons
☐	☒	A full description of the statistical parameters including central tendency (e.g. means) or other basic estimates (e.g. regression coefficient) AND variation (e.g. standard deviation) or associated estimates of uncertainty (e.g. confidence intervals)
☐	☒	For null hypothesis testing, the test statistic (e.g. *F*, *t*, *r*) with confidence intervals, effect sizes, degrees of freedom and *P* value noted<br>*Give P values as exact values whenever suitable.*
☒	☐	For Bayesian analysis, information on the choice of priors and Markov chain Monte Carlo settings
☒	☐	For hierarchical and complex designs, identification of the appropriate level for tests and full reporting of outcomes
☐	☒	Estimates of effect sizes (e.g. Cohen's *d*, Pearson's *r*), indicating how they were calculated

Our web collection on statistics for biologists contains articles on many of the points above.

Software and code

Policy information about availability of computer code

Data collection	Data collection was of standard BAM files and raw methylation levels
Data analysis	Ancient methylation was computed using RoAM, which was reported in a previous study (Gokhman et al, Science 2014) and is distributed freely from http://carmelab.huji.ac.il/software.html

For manuscripts utilizing custom algorithms or software that are central to the research but not yet described in published literature, software must be made available to editors/reviewers. We strongly encourage code deposition in a community repository (e.g. GitHub). See the Nature Research guidelines for submitting code & software for further information.

Data

Policy information about availability of data

All manuscripts must include a data availability statement. This statement should provide the following information, where applicable:

- Accession codes, unique identifiers, or web links for publicly available datasets
- A list of figures that have associated raw data
- A description of any restrictions on data availability

All sequencing and methylation data generated in this work have been deposited in NCBI's Gene Expression Omnibus under GEO accession number GSE96833 [https://www.ncbi.nlm.nih.gov/geo/query/acc.cgi?acc=GSE96833]. All other data and materials are contained in the paper and its supplementary information or available upon request.

Field-specific reporting

Please select the one below that is the best fit for your research. If you are not sure, read the appropriate sections before making your selection.

Ecological, evolutionary & environmental sciences study design

All studies must disclose on these points even when the disclosure is negative.

Study description	Comparative analysis of DNA methylation maps across modern human, archaic humans and chimpanzees
Research sample	DNA methylation in three archaic humans, five ancient anatomically modern humans, 58 modern human bones, and six chimpanzees
Sampling strategy	All publically avaialble bone DNA methylation maps, all high coverage and UDG treated archaic genomes
Data collection	Most data is publically available. Modern human bones were collected under Helsinki approval #0178-13-HMO
Timing and spatial scale	Recent human evolution, roughly the last 600,000 years
Data exclusions	No data were excluded
Reproducibility	Not relevant
Randomization	Permutation tests were performed as described in Methods
Blinding	Not relevant

Did the study involve field work? ☐ Yes ☒ No

Reporting for specific materials, systems and methods

We require information from authors about some types of materials, experimental systems and methods used in many studies. Here, indicate whether each material, system or method listed is relevant to your study. If you are not sure if a list item applies to your research, read the appropriate section before selecting a response.

Materials & experimental systems

n/a	Involved in the study	
☒	☐	Antibodies
☒	☐	Eukaryotic cell lines
☒	☐	Palaeontology
☐	☒	Animals and other organisms
☐	☒	Human research participants
☒	☐	Clinical data

Methods

n/a	Involved in the study	
☒	☐	ChIP-seq
☒	☐	Flow cytometry
☒	☐	MRI-based neuroimaging

Animals and other organisms

Policy information about studies involving animals; ARRIVE guidelines recommended for reporting animal research

Laboratory animals	None
Wild animals	We used bones of deceased chimpanzees. This includes a 47-year-old female chimpanzee provided from the Biobank of the Biomedical Primate Research Centre (BPRC) in the Netherlands, a newborn wild chimpanzee infant who died during a documented infanticide event at Gombe National Park on 9 March 2012, and four chimpanzee cadavers from captive colonies at the Southwest National Primate Research Center in Texas, USA. All this is described in the paper.
Field-collected samples	See above
Ethics oversight	None

Note that full information on the approval of the study protocol must also be provided in the manuscript.

Human research participants

Policy information about studies involving human research participants

Population characteristics	Standard knee and hip replacement surgeries
Recruitment	Only signed consent

Ethics oversight	Helsinki approval #0178-13-HMO

Note that full information on the approval of the study protocol must also be provided in the manuscript.
