# Supplementary material for: Differential DNA methylation of vocal and facial anatomy genes in modern humans
Source: Nat Commun. 2020 Mar 4;11:1189. doi: 10.1038/s41467-020-15020-6 (PMC7055320; doi:10.1038/s41467-020-15020-6)
Supplement: Supplementary file 12 — Description of Additional Supplementary Files [file 41467_2020_15020_MOESM12_ESM.pdf]

Title: Supplementary Data 1.

Description: ('Samples' sheet) Ancient and present-day DNA methylation samples. ('Chimpanzee RRBS' sheet) Statistics for chimp bone RRBS.

Title: Supplementary Data 2.

Description: ('AMH/Archaic/Neanderthal/Denisovan-derived' sheets) DMRs identified between the human groups, ranked according to their level of significance. ('Chimpanzee-human diffs' sheet) DMRs identified between chimpanzee and human samples.

Title: Supplementary Data 3.

Description: ('GO terms' sheet) Significant gene ontology terms (biological process) in AMH-derived DMGs. ('UniGene expression' sheet) UNIGENE Expression enrichment values of AMH-derived DMGs. ('UP_Tissue exp. LowVar' sheet) UP_Tissue Expression enrichment values of AMH-derived DMGs with low inter-tissue variability. ('GNF exp. LowVar' sheet) GNF Expression enrichment values of AMH-derived DMGs with low inter-tissue variability. ('UP_Tissue expression' sheet) UP_Tissue Expression enrichment values of AMH-derived DMGs. ('GNF expression' sheet) GNF Expression enrichment values of AMH-derived DMGs.

Title: Supplementary Data 4.

Description: ('AMH-derived' sheet) Body parts significantly enriched (FDR < 0.05) within AMH-derived DMGs. ('topQuartile AMH-derived' sheet) Body parts significantly enriched (FDR < 0.05) within top quartile AMH-derived DMGs. ('AMH-derived inc. RRBS' sheet) Body parts significantly enriched (FDR < 0.05) within AMH-derived DMGs, which were also filtered using the RRBS samples (infant chimpanzee and juvenile AMH). ('Archic-derived' sheet) Body parts significantly enriched (FDR < 0.05) within Archaic-derived DMGs. ('AMH-derived rem.Osteogenic diff' sheet) Body parts significantly enriched (FDR < 0.05) within AMH-derived DMGs, after removal of 20 DMRs found <10kb from loci where methylation was shown to change during osteogenic differentiation. ('Neanderthal-derived, as AMH' sheet) Enrichment levels of body parts within Neanderthal-derived DMGs, ran as Ust'-Ishim-derived DMGs to test whether the filtering process results in enrichment of the vocal cords, larynx or facial regions. ('Denisovan-derived, as AMH' sheet) Enrichment levels of body parts within Denisovan-derived DMGs, ran as Ust'-Ishim-derived DMGs to test whether the filtering process results in enrichment of the vocal cords, larynx or facial regions. ('Ust'-Ishim-derived, as Archaic' sheet) Enrichment levels of body parts within Ust'-Ishim-derived DMGs, ran as Neanderthal/Denisovan-derived DMGs to test whether the filtering process results in enrichment of the vocal cords, larynx or facial regions.

Title: Supplementary Data 5.

Description: ('Face' sheet) DMRs in genes affecting the facial skeleton. ('Larynx' sheet) DMRs in genes affecting the larynx and vocal tract.

Title: Supplementary Data 6.

Description: ('AMH-derived' sheet) The fraction of differentially methylated CpGs in windows of 100 kb around each DMR. Shown is the mean fraction in DMGs associated with each body part. ('AMH-derived (skeletal)' sheet) The fraction of differentially methylated CpGs in windows of 100 kb

around each skeleton-related DMR. Shown is the mean fraction in DMGs associated with each body part. (‘Archaic-derived’ sheet) The fraction of differentially methylated CpGs in windows of 100 kb around each DMR. Shown is the mean fraction in DMGs associated with each body part.

Title: Supplementary Data 7.

Description: Skeleton-related genes, and the number and fraction of HPO phenotypes that are divergent between Neanderthals and anatomically modern humans.

Title: Supplementary Data 8.

Description: (‘Marshall Smith vs. Neanderthal’ sheet) Phenotypes associated with the Marshall-Smith syndrome and their directionality in the Neanderthal. Non-directional phenotypes (such as irregular dentition), or phenotypes that are pronounced in both directions (some patients have a short stature, some have a tall stature) were discarded. (‘Malan vs. Neanderthal’ sheet) Phenotypes associated with the Malan syndrome and their directionality in the Neanderthal. Non-directional phenotypes (such as irregular dentition), or phenotypes that are pronounced in both directions (some patients have a short stature, some have a tall stature) were discarded.
